# Tumor irradiation promotes antigen dressing of dendritic cells to enhance CAR T cell persistence and efficacy in lung metastases

**DOI:** 10.1038/s43018-026-01167-6

**Published:** 2026-05-22

**Authors:** Sophia Navarre, Maki N. Ishibashi, Achuth Nair, Ivan Reyes-Torres, Meriem Belabed, Laszlo Halasz, Matthew D. Park, Raphaël Mattiuz, Merouane Ounadjela, Gertrude Gunset, Jorge Mansilla-Soto, Judith Feucht, Annalisa Cabriolu, Jessica Le Berichel, Alexander Birbrair, Justin Eyquem, Brian D. Brown, Miriam Merad, Michel Sadelain, Jalal Ahmed

**Affiliations:** 1 Department of Immunology and Immunotherapy, Marc and Jennifer Lipschultz Precision Immunology Institute, Icahn School of Medicine at Mount Sinai, New York, NY USA; 2 Department of Medicine, Immunology Program, Gene Transfer and Somatic Cell Engineering Laboratory, Center for Cell Engineering and Immunology Program, Sloan Kettering Institute, Memorial Sloan Kettering Cancer Center, New York, NY USA; 3 Department of Dermatology, University of Wisconsin-Madison, Madison, WI USA; 4 Department of Medicine—Division of Hematology and Oncology, Gladstone-UCSF Institute of Genomic Immunology, Parker Institute for Cancer Immunotherapy, University of California, San Francisco, CA USA; 5 Human Immune Monitoring Center, Icahn School of Medicine at Mount Sinai, New York, NY USA; 6 Columbia Initiative in Cell Engineering and Therapy (CICET), Cancer Cell Therapy Initiative in the Vagelos Physicians and Surgeons, Columbia University Irving Medical Center, New York, NY USA; 7 Department of Radiation Oncology, Icahn School of Medicine at Mount Sinai, New York, NY USA; 8 Present Address: Department of Biomedical Engineering, The City College of New York, New York, NY USA; 9 Present Address: Medical Scientist Training Program, University of Pittsburgh School of Medicine, Pittsburgh, PA USA; 10 Present Address: Louis V. Gerstner Jr. Graduate School of Biomedical Sciences, Memorial Sloan Kettering Cancer Center, New York, NY USA; 11 Present Address: Columbia Initiative in Cell Engineering and Therapy (CICET), Cancer Cell Therapy Initiative in the Vagelos Physicians and Surgeons, Columbia University Irving Medical Center, New York, NY USA; 12 Present Address: Department of Immunology, H. Lee Moffitt Cancer Center and Research Institute, Tampa, FL USA; 13 Present Address: Cluster of Excellence iFIT (EXC2180) ‘Image-guided and Functionally Instructed Tumor Therapies’, University Children’s Hospital Tübingen, Tübingen, Germany

**Keywords:** Cancer immunotherapy, Tumour immunology, Cancer

## Abstract

Metastatic solid tumors remain the principal cause of cancer mortality worldwide. High tumor burden impairs responses to chimeric antigen receptor (CAR) T cell therapy, yet off-tumor toxicity limits the doses that can be safely delivered. Strategies to selectively enhance CAR T cell activity at tumor sites could widen the therapeutic window. Using syngeneic models of extensive metastatic lung adenocarcinoma and melanoma, we show that 8 Gy of tumor irradiation significantly enhanced CAR T cell persistence in a manner critically dependent on dendritic cells (DCs). Irradiation promoted trogocytic antigen dressing of tumor antigens onto DCs, which then expanded CAR T cells through the chimeric receptor. Without functional DCs, irradiation failed to sustain CAR T cell persistence and tumors relapsed. Irradiation increased CAR T cell numbers within tumors but not in adjacent normal lung tissue that also expressed target antigen, conferring robust control of tumor without increased toxicity. These data define a mechanistic basis and rationale for combining radiotherapy with CAR T cell therapy.

## Abstract (web summary)

Ahmed and colleagues show that, by promoting ‘dressing’ of intact tumor target antigens onto dendritic cells, tumor irradiation enhances chimeric antigen receptor T cell persistence and efficacy in lung metastasis models.

## Main

CARs are engineered receptors that redirect the specificity of T cells to target antigens^1,2^. CAR T cells directed to the target antigen CD19 can clear large volumes of B cell lymphoma^3^, often achieving durable remissions. This discovery ignited efforts to apply CAR technologies to other advanced cancers where treatment options remain limited^4,5^. In stark contrast to CD19 CAR T cells, however, CAR T cells targeted to solid tumors have diminished expansion and do not persist^4,5^.

This is attributed in part to the immunosuppressive microenvironment within solid tumors, a challenge that is compounded by the extensive tumor burden common in participants enrolled in CAR T cell trials and the narrow therapeutic window when targeting antigens shared with normal tissues. Fatal on-target, off-tumor toxicity has been reported with infusion of 1 × 10^10^ ERBB2 CAR T cells^6^. Conversely, doses up to 3 × 10^8^ CAR T cells per m^2^ of mesothelin CAR T cells did not confer radiographic responses and the CAR transgene was undetectable in autopsy specimens^7^. Despite a rapid evolution of technologies that venture to optimize the CAR T cell product^8^, few strategies have effectively addressed the inherent limitations of this narrow therapeutic window in treating extensive solid cancers or the profound negative influence of the tumor microenvironment (TME)^9^. No existing strategies enable controlled CAR T cell expansion at the tumor site.

Crucially, the chimeric receptor can provide a means for T cells to derive costimulatory signals from antigen-presenting cells (APCs) labeled with the CAR target. DCs, the most potent APCs at driving T cell responses, have successfully been engaged to expand CAR T cells. One approach enlists the endogenous T cell receptor (TCR) of virus-specific T cells to recognize viral peptides presented on the major histocompatibility complex (MHC) by DCs^10,11^. Alternatively, RNA-LPX and Amph-ligand technologies that mark DCs with target antigens in vivo can expand CAR T cells through the chimeric receptor^12,13^—a promising approach being tested in a phase 1 clinical trial for solid tumors (NCT04503278)^14^. In the absence of vaccination-based approaches, the limited persistence of solid tumor-directed CAR T cells suggested that APC-derived costimulatory signals are inaccessible in this setting and no role for DCs was apparent. However, we observed that syngeneic 28ζ CAR T cells had enhanced efficacy when directed to ectopic and endogenous antigens on irradiated tumors and aimed to resolve how the immunology of CAR T cells differs in this context.

By targeting a cross-species homologous antigen (human CD19) not expressed by the host with a mouse scFv (SJ25C1 mouse IgG1κ^15^) that does not bind endogenous host antigens, we intended to isolate the ‘on-target’ activity of CAR T cells to target^+^ tumor cells. However, we discovered that antigen dressing of tumor antigens onto DCs enabled these potent APCs to expand T cells through the chimeric receptor. We quantify the effect of tumor irradiation on CAR T cell infiltration and cytotoxicity, as well as on antigen dressing by DCs, and explore whether tumor irradiation can widen the therapeutic window of CAR T cell therapy for persons with extensive solid tumor, even when targeting tumor antigens also expressed endogenously on normal tissues.

## Results

**Extended Data Fig. 1:**
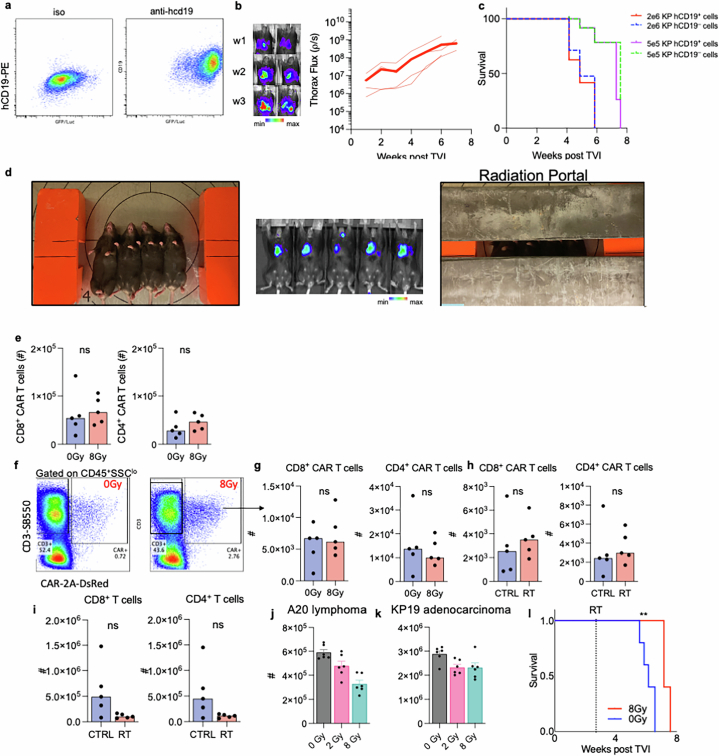
Generating a non-immunogenic lung tumor model targeted by CAR T cells. **a**, Flow cytometry of hCD19-expressing KP lung adenocarcinoma clone expressing GFP-Luciferase. **b**, Bioluminescence kinetics of KP-hCD19^+^ GFP-Luciferase^+^ clonal cell line after tail-vein injection into C57BL/6 mice, quantified in right panel, n = 4 mice. **c**, Survival of mice after tail-vein injection (TVI) of KP lung adenocarcinoma cells expressing the CAR target hCD19 compared to founder KP clones that are negative for hCD19 expression (Target^*−*^). Two dose levels were used as indicated. Expression of hCD19 did not alter growth kinetics of KP tumor cells suggesting that this human gene did not induce an endogenous adaptive immune response. n = 5 mice per group. **d**, Irradiation protocol. Middle figure showed bioluminescence imaging of KP-hCD19^+^ GFP-Luciferase^+^ clonal cell line three weeks after tail-vein injection into C57BL/6 mice, illustrating that the majority of lung tumor bioluminescence is captured within the “radiation portal” shown in the right panel. Anesthetized mice were placed supine on an aluminum shelf plate under a cone shaped irradiation field (left panel) in a RS2000 Small Animal Irradiator (Rad Source Technologies Inc.) to deliver 160 kV photons at a 25 mA, with a dose rate of 2.1 Gy/min. Mice were treated with a half-beam block technique (right panel) eliminating divergence into the neck superiorly. Radiation was delivered to the thorax through an aperture formed between two 2 mm lead sheets placed above the mice, that shielded the head and neck superior to the apex of the thorax, and tissues inferior to the xiphoid process. **e-g**, Quantification of numbers of CD8^+^ and CD4^+^ CAR T cells in the lungs (**e**) or thoracic LNs (**f,g**) of KP19-tumor bearing mice harvested 3 days after adoptive transfer of CAR T cells. n = 5 mice per group, two-sided t-test. **h-i**, numbers of CD8^+^ and CD4^+^ CAR T cells (**h**) or endogenous T cells (**i**) in thoracic LNs of KP19-tumor bearing mice harvested 9 days after adoptive transfer of CAR T cells. n = 5 mice per group, two-sided t-test. **j-k**, Quantification of numbers of A20 (**j**) or hCD19^+^ KP cells (**k**) 2 days after plating. Cells were exposed to indicated doses of irradiation, or not, 24 hours prior to harvesting (KP cells trypsinized) and quantification. n = 6 technical replicates, mean ± SEM. Results representative of three independent experiments. **l**, Survival of mice after TVI 5×10^5^ of hCD19^+^ KP lung adenocarcinoma cells. Mice were randomized to treatment with 8 Gy of thoracic irradiation or not, then followed for survival. TVI, tail vein injection; RT, radiotherapy. n = 5 mice per group. Logrank Mantel-Cox test ** p value 0.0027. Source data

To model the immune cell interactions that CAR T cells encounter within the TMEs of individuals, we use a syngeneic *Kras*^G12D^;*Trp53*^−/−^ (KP) lung tumor model. This model has been shown by 10x single-cell sequencing to develop myeloid infiltrates akin to those seen in human non-small-cell lung cancer (NSCLC)^16,17^. Moreover, to model the treatment of extensive solid cancers, we administer CAR T cells to orthotopic lung tumors 28 days after tail-vein injection (TVI), when KP tumors measure 2.2 mm^2^ on average and occupy 20% of lung area^17^. Untreated mice typically reach humane endpoints within the following 2 weeks (Extended Data Fig. 1a–c,l), reflecting the advanced stage of the disease at the time of CAR T cell treatment.

### Irradiation enhances CAR T cell persistence and efficacy against established lung tumors

Clonal KP cell lines transduced with the model target antigen human CD19 (hCD19) developed with identical kinetics to founder lines (Extended Data Fig. 1a–c), suggesting that this antigen did not trigger an endogenous immune response that measurably impacted tumor growth.

Tail vein-injected KP cells engraft primarily within the lungs, enabling us to irradiate them in a thoracic radiotherapy (TRT) portal (Extended Data Fig. 1d). Furthermore, 8 Gy of TRT does not cause radiation-induced lung injury or impact survival of wild-type (WT) C57BL/6J mice^18^ and is a clinically well-tolerated palliative dose^19^.

**Fig. 1:**
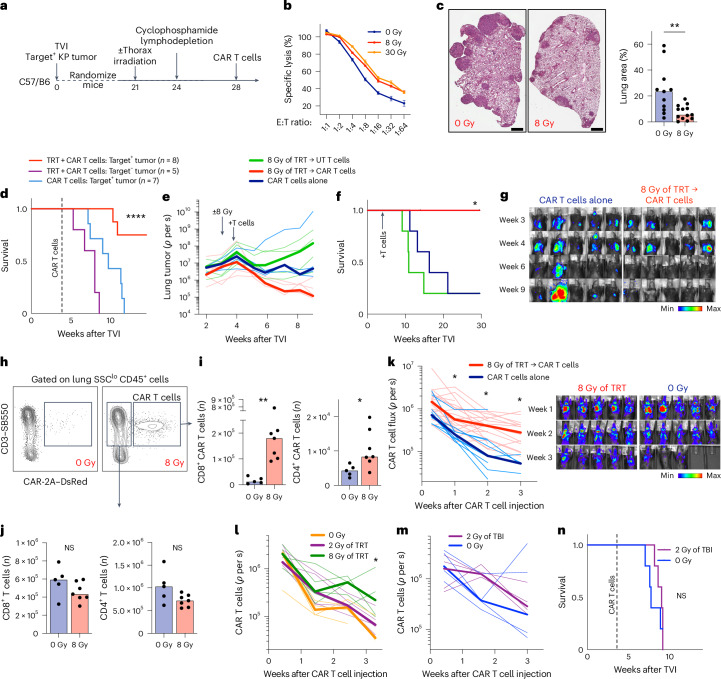
Enhanced persistence and efficacy of CAR T cells in irradiated tumors. **a**,**c**,**d**, C57BL/6 female mice inoculated with 5 × 10^5^ target^+^ (hCD19^+^) orthotopic lung tumors (KP19) through TVI were randomized 3 weeks later to 8 Gy of TRT or no irradiation. Cohorts were lymphodepleted and treated with syngeneic T cells transduced with a h1928ζ-2A-DsRed retroviral construct to a dose of 4 × 10^6^ CAR T cells per mouse and lungs were isolated 9 days after CAR T cell transfer. **b**, Target^+^ Luciferase^+^ KP19 cells were irradiated at the doses indicated before trypsinization 8 h later for coculture at indicated effector-to-target (E:T) ratios with CAR T cells. Data are mean ± s.e.m. (*n* = 12 technical replicates per dilution). **c**, Left: representative H&E images of tumor-bearing lungs of mice on day 9 after CAR T cell therapy. Scale bar, 1 mm. Right: quantification of tumor area as a percentage of the total area of the lung cross-section (right). Data from two experiments; each dot represents a mouse (*n* = 11 mice for 0 Gy, *n* = 13 mice for 8 Gy). *P* = 0.0041, **P < 0.01, two-sided *t*-test. **d**, Survival of mice injected with 5 × 10^5^ target^+^ or target^*−*^ KP cells per mouse. After 3 weeks, mice were irradiated or not, lymphodepleted and treated with 2.5 × 10^6^ CAR T cells per mouse. ****P < 0.0001, log-rank (Mantel–Cox) test. **e**–**g**, Thoracic bioluminescence kinetics (**e**,**g**) and survival (**f**) of mice injected with 5 × 10^5^ CBR-Luciferase^+^ target^+^ KP cells (**e**–**g**). Mice were randomly allocated to treatment groups based on thoracic tumor luminescence at 2 weeks after TVI of tumor cells as in **a** and lymphodepleted and treated with untransduced (UT) or CAR-transduced syngeneic mouse T cells. Faint lines track tumor bioluminescence from individual mice. Median values are in bold (*n* = 5). *P = 0.0259, log-rank (Mantel–Cox) test. **h**–**j**, Representative flow cytometry plots (**h**) and numbers of DsRed^+^ CAR T cells (**i**) or DsRed^*−*^CD45^+^CD3^+^ endogenous T cells (**j**) in lungs of KP19 tumor-bearing female mice 9 days after adoptive transfer of CAR T cells. Mice inoculated with 5 × 10^5^ target^+^ KP lung tumors were treated with syngeneic T cells transduced with a CAR-2A–DsRed retroviral construct to a dose of 4 × 10^6^ CAR T cells per mouse (*n* = 5 mice for 0 Gy, *n* = 7 mice for 8 Gy). **P = 0.0025 (left) and *P = 0.0177 (right), Mann–Whitney *U* test. Data representative of duplicate experiments. NS, not significant. **k**–**m**, Thoracic bioluminescence kinetics of Luciferase and CAR double-transduced syngeneic mouse T cells (2.5 × 10^6^ CAR T cells per mouse) injected into mice inoculated with 5 × 10^5^ target^+^ orthotopic KP lung tumors as in **a**. Data in **k** were combined from two independent experiments (*n* = 10 for 0 Gy, *n* = 20 for 8 Gy). *P < 0.05; week 1, *P* = 0.0332; week 2, *P* = 0.0211; week 3, *P* = 0.0329, two-sided *t*-test. Data in **l**,**m**, *n* = 5. Faint lines track T cell bioluminescence from individual mice. Median values are in bold. *P* = 0.0190, *P < 0.05, ordinary one-way ANOVA. **n**, Survival of mice injected with 5 × 10^5^ target^+^ KP cells followed by indicated treatment before infusion of 2.5 × 10^6^ CAR T cells per mouse (*n* = 5 per group). Statistical analysis conducted using a log-rank (Mantel–Cox) test. Source data

We then assessed whether irradiation enhanced CAR T cell cytotoxicity (Fig. 1a–c). We found that 8 Gy sensitized target^+^ KP cells to CAR T cell cytotoxicity, with no further benefit from higher doses (Fig. 1b). C57BL/6 lung-tumor-bearing mice were treated with either 8 Gy of TRT (Extended Data Fig. 1d) or left unirradiated before lymphodepletion with cyclophosphamide and adoptive transfer of syngeneic CAR T cells^20^ (Fig. 1a). Irradiated mice showed significantly reduced tumor burden (Fig. 1c), indicating enhanced CAR T cell cytotoxicity in vivo. Furthermore, CAR T cells targeted to irradiated target^+^ tumors but not target^*−*^ tumors significantly increased survival (Fig. 1d), demonstrating a requirement for both tumor irradiation and CAR target recognition by T cells for effective treatment.

Tracking of thoracic tumor bioluminescence revealed that both CAR T cells alone and irradiation without CAR T cells temporarily reduced tumor bioluminescence. However, only mice that received irradiation followed by CAR T cell treatment showed a pronounced and sustained reduction in tumor bioluminescence (Fig. 1e,g) and improved survival (Fig. 1f).

These data suggested that irradiation enhanced CAR T cell efficacy by sensitizing tumors to cytotoxicity. Notably, tumor bioluminescence declined steadily (Fig. 1e), indicating prolonged CAR T cell activity lasting several weeks. These data compelled us to explore whether CAR T cells were behaving differently in irradiated tumors.

Lung-tumor-bearing mice were treated with syngeneic T cells transduced with CAR–2A–DsRed constructs and the number of DsRed^+^ CAR T cells was quantified in dissociated PBS-perfused lungs. Irradiation did not impact homing, as the number of CAR T cells was similar in the lungs and thoracic lymph nodes of irradiated and unirradiated mice 3 days after injection (Extended Data Fig. 1e–g). However, 9 days after transfer, the numbers of CD8^+^ and CD4^+^ CAR T cells were eightfold and twofold higher, respectively, in irradiated tumor-bearing lungs (Fig. 1h,i) but not in lymph nodes (Extended Data Fig. 1h), indicating that irradiation enhanced CAR T cell numbers where tumors were. Notably, DsRed^*−*^CD45^+^CD3^+^ endogenous T cells were not expanded in either lungs or lymph nodes in irradiated mice (Fig. 1j and Extended Data Fig. 1i), suggesting that these cells did not contribute significantly to the antitumor response.

To track CAR T cell numbers in individual mice, lung-tumor-bearing mice were treated with syngeneic T cells double-transduced with CAR and click beetle red (CBR) Luciferase constructs^20^. CAR T cell bioluminescence declined rapidly in unirradiated mice. In irradiated mice, however, CAR T cell bioluminescence diverged after the first week and remained elevated (Fig. 1k). Evidently, the effect of irradiation was twofold: increasing cytotoxicity and enhancing CAR T cell persistence.

Low-dose radiotherapy delivered focally^21^ or to the entire host (total-body irradiation, TBI)^22^ has been shown to enhance CAR T cell activity against lymphoma. Lymphoid lineages, however, are sensitive to irradiation^23^, while human NSCLC lineages are among the most radioresistant^24^. Unlike A20 lymphoma cells, KP adenocarcinoma cells were resistant to increasing doses of irradiation (Extended Data Fig. 1j,k). Moreover, 8 Gy of TRT alone marginally delayed progression of disease (Extended Data Fig. 1l).

To test whether low-dose irradiation could impact CAR T cell activity, target^+^ KP lung-tumor-bearing mice were treated with 2 Gy or left unirradiated before CAR T cell treatment as before. Low-dose TRT did not increase the persistence of CAR T cells to the same degree as 8 Gy (Fig. 1l). Interestingly, 2 Gy of TBI transiently increased the persistence of CAR T cells compared to unirradiated mice but did not impact survival (Fig. 1m,n), suggesting that higher irradiation doses are required to enhance CAR T cell efficacy against radioresistant histologies such as lung adenocarcinoma.

### Tumor irradiation enhances CAR T cell effector function

**Fig. 2:**
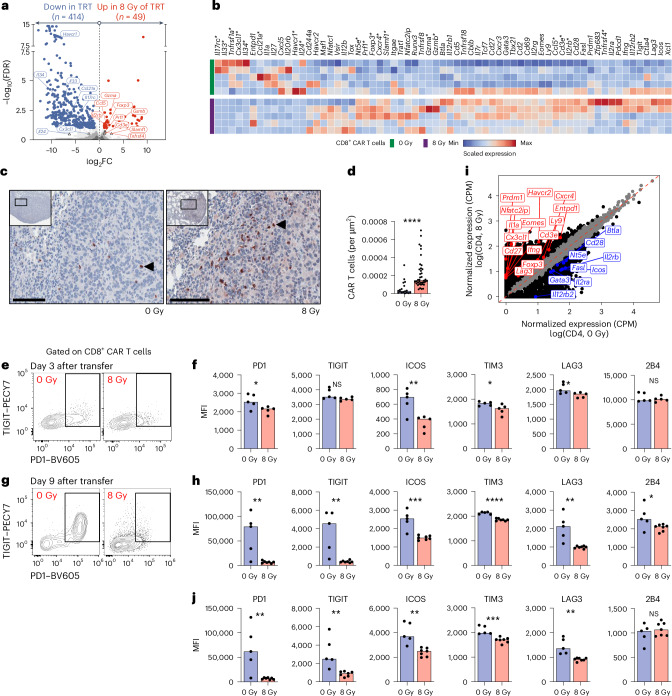
Tumor irradiation induces transcriptional programs and phenotype associated with enhanced CAR T cell effector functions. **a**,**b**, CD8^+^ CAR T cells were sorted from single-cell suspensions of tumor-bearing lungs isolated 10–12 days after adoptive transfer (as in Fig. 1a) and individually processed for bulk sequencing (*n* = 5). **a**, Volcano plot of DEGs (FDR < 0.1 and FC > 1) in CD8^+^ CAR T cells from irradiated versus unirradiated mice. **b**, Heat map displaying relative amounts of transcripts of genes clustered by expression (columns) and mice (rows). Rows represent individual mice (*n* = 5 mice per group). Relative expression levels (*z* scores) of genes are shown, color-coded according to legend. Asterisks indicate significant DEGs (two-sided Wald test, *P* < 0.05). **c**, Representative paraffin sections of KP19 tumor-bearing lungs isolated 9 days after adoptive transfer and stained for DsRed expression on CAR T cells (hematoxylin counterstain). Black arrowheads indicate DsRed^+^ CAR T cells. Scale bars, 100 µm. **d**, Quantification of intratumoral DsRed^+^ CAR T cell density. Each dot represents a tumor nodule from representative lung sections (*n* = 5 mice for 0 Gy (18 tumors), *n* = 7 mice for 8 Gy (40 tumors)). ****P < 0.0001, two-sided Mann–Whitney *U*-test. **e**–**h**, Representative cytometry plots (**e**,**g**) and quantification (**f**,**h**) of mean fluorescence intensity (MFI) of indicated surface markers on CD8^+^ CAR T cells 3 days (**e**,**f**; *n* = 5 mice; two-sided *t*-test: PD1, *P* = 0.0388; TIGIT, *P* = 0.076; ICOS, *P* = 0.0071; TIM3, *P* = 0.0425; LAG3, *P* = 0.039; 2B4, *P* = 0.4899) or 9 days (**g**,**h**; *n* = 5 mice for 0 Gy, *n* = 7 mice for 8 Gy; two-sided *t*-test: PD1, *P* = 0.0046; TIGIT, *P* = 0.003; ICOS, *P* = 0.0005; TIM3, *P* < 0.0001; LAG3, *P* = 0.0023; 2B4, *P* = 0.0491) after adoptive transfer. Data are representative of an experiment performed in duplicate. **i**, CD4^+^ CAR T cells were sorted from single-cell suspensions of tumor-bearing lungs isolated 10–12 days after adoptive transfer (as in Fig. 1a) and pooled (*n* = 5 mice per group) before processing for bulk sequencing. Genes with <twofold expression between groups are shown in gray; others are shown in black. **j**, Quantification of MFI of indicated surface markers on CD4^+^ CAR T cells 9 days after adoptive transfer (*n* = 5 mice for 0 Gy, *n* = 7 mice for 8 Gy; two-sided *t*-test: PD1, *P* = 0.0057; TIGIT, *P* = 0.0042; ICOS, *P* = 0.0013; TIM3, *P* = 0.0006; LAG3, *P* = 0.0014; 2B4, *P* = 0.3759). *P < 0.05, **P < 0.01. Source data

To identify gene signatures associated with enhanced CAR T cell activity, CAR T cells were sorted for RNA sequencing from irradiated and unirradiated lung-tumor-bearing mice 2 weeks after transfer. A total of 522 genes were differentially expressed (DESeq2, *n* = 5 per group, FDR < 0.1; Fig. 2a and Supplementary Table 1), of which 71 genes were enriched in CD8^+^ CAR T cells isolated from irradiated mice compared to unirradiated controls, while 451 genes showed significantly lower expression.

**Extended Data Fig. 2:**
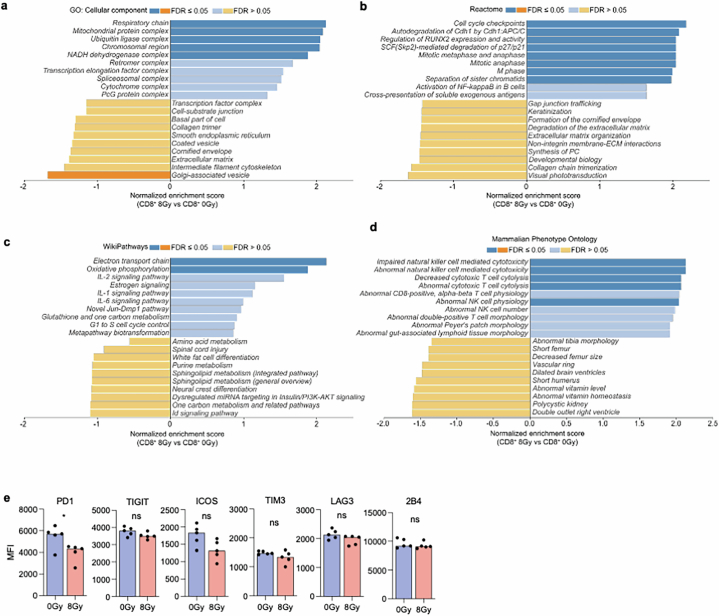
Differential transcriptional programs and phenotypes of CAR T cell incident on irradiated tumors. **a-d**, 1589 genes that were differentially expressed between CD8^+^ CAR T cells sorted from irradiated vs unirradiated mice (DESeq2, threshold of p < 0.05) were used for gene set enrichment analysis using WebGestalt. 5 mice per group. **e**, Quantification of mean fluorescence intensity (MFI) of indicated surface markers on CD4^+^ CAR T cells 3-days post adoptive transfer. n = 5 mice per group, two-sided t-test * p value 0.0332. Source data

Tumor irradiation promoted expression of genes involved in ‘respiratory chain’, ‘mitochondrial protein complex’ and ‘NADH dehydrogenase complex’ Gene Ontology cell components (Extended Data Fig. 2a) and cell-cycle pathways (Extended Data Fig. 2b). WikiPathways ontology terms showed significant enrichment in ‘electron transport chain’, as well as enrichment of pathways involved in ‘oxidative phosphorylation’ and ‘IL-2 signaling’ (Extended Data Fig. 2c). These analyses suggest that CD8^+^ CAR T cells exhibit differential metabolic activity and cytokine signaling in irradiated tumors.

We then assessed expression genes broadly implicated in T cell immunology (Fig. 2a,b). CD8^+^ CAR T cells in irradiated tumors demonstrated elevated expression of the transcription factor *Foxp3*, previously shown to be expressed in effector CD8^+^ T cells within human NSCLC where it was associated with higher levels of granzymes and effector cytokines^25^. Consistent with this, CD8^+^ CAR T cells in irradiated tumors were enriched in mammalian phenotype ontology terms involved in cytolysis (Extended Data Fig. 2d) and key effector genes used by cytotoxic T cells to induce apoptosis in target cells (*Prf1*, *Gzma*, *Gzmb* and *Rab27a*) (Fig. 2a,b), indicating that these cells were poised for cytotoxic function. Moreover, the number of tumor-infiltrating CAR T cells was fivefold greater in irradiated mice (Fig. 2c,d), reflecting an elevated effector-to-target ratio. Along with increased expression of cytotoxicity-related and metabolism-related genes, this suggested that the enhanced cytotoxicity observed in vivo (Fig. 1c,e) was not solely because of tumor cell sensitization.

Although gene transcripts associated with T cell dysfunction and exhaustion had variable expression (Fig. 2a,b), surface levels of checkpoint and coinhibitory receptors were elevated on CD8^+^ CAR T cells as early as 3 days after transfer into unirradiated mice (Fig. 2e,f) and continued to rise the following week (Fig. 2g,h). However, surface levels of these receptors remained low on CAR T cells in irradiated mice. Similarly, CD4^+^ CAR T cells in irradiated mice did not vary in levels of checkpoint or coinhibitory receptor transcripts (Fig. 2i) but maintained low surface expression of these receptors (Fig. 2j and Extended Data Fig. 2e). These data suggest that CAR T cells in irradiated tumors downregulate coinhibitory receptors post-transcriptionally.

### Anti-CSF1R preconditioning fails to enhance CAR T cell efficacy

**Extended Data Fig. 3:**
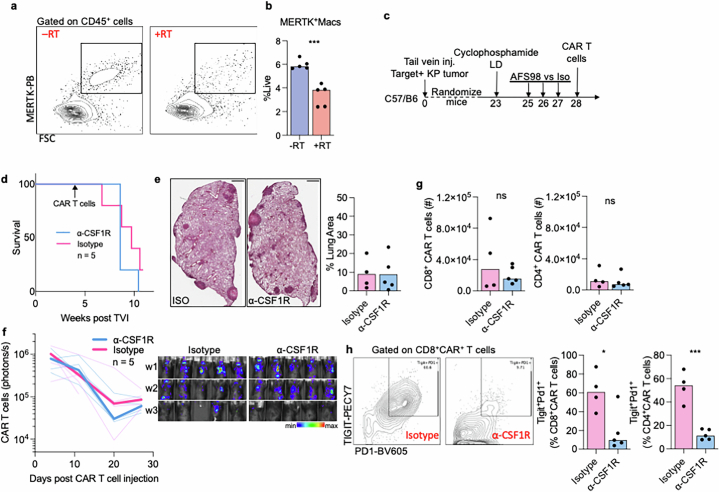
α-CSF1R preconditioning does not enhance CAR T cell persistence or therapeutic efficacy. **a-b**, Representative flow cytometry plots and quantification (**b**) of MERTK^+^ macrophages in KP tumor-bearing lungs. Mice inoculated with via tail vein-injection of 5×10^5^ tumor cells were randomized three weeks later to 8 Gy thoracic irradiation or no irradiation. Mice were then harvested 2 weeks after radiotherapy and macrophages were quantified. n = 5 mice per group. Two-tailed t-test *** p value 0.0004. **c-h**, Mice inoculated with 5×10^5^ target^+^ orthotopic KP lung tumors via tail vein-injection were lymphodepleted and randomized three weeks later to treatment with anti-CSF1R or Isotype antibody prior to CAR T cell i.v. injection. Results representative of two independent experiments. **d**, Survival of mice treated with anti-CSF1R or Isotype antibody prior to CAR T cell i.v. injection. Log rank (Mantel-Cox) test. **e**, Representative H&E images of tumor-bearing lungs of mice at day 9 after CAR T cell therapy treated with either α-CSF1R or Isotype antibody. Quantification of the tumor area as a percent of the total area of the lung cross-section (right) is shown. n = 4 (Isotype) n = 5 (aCSF1R) mice per group. Scale bar = 1 mm. **f**, Kinetics of thoracic CAR T cell persistence by Bioluminescence imaging. Mice inoculated with KP19 lung tumors were lymphodepleted and randomized to treatment with anti-CSF1R or Isotype antibody as in **c**, and then treated with Luciferase and CAR double-transduced syngeneic mouse T cells. Faint lines track BLI from individual mice, median values shown in bold. n = 5. **g**, Quantification of CD8^+^ and CD4^+^ DsRed^+^ CAR T cell numbers in enzymatically digested lungs of KP19-tumor bearing mice harvested 9 days after adoptive transfer of CAR T cells treated with either α-CSF1R or Isotype antibody. n = 4 (Isotype) n = 5 (aCSF1R) mice per group, two-sided t-test. **h**, Representative flow cytometry plots and quantification of CD8^+^ and CD4^+^ CAR T cells isolated 9-days post transfer and stained for expression of indicated surface markers. n = 4 (Isotype) n = 5 (aCSF1R) mice per group; two-tailed t-test * p value 0.0185, *** p value 0.0003. RT, radiotherapy, LD, lymphodepletion. Source data

Macrophages are the predominant myeloid population within the TME of human NSCLC^26^ and have recently been shown to exhibit the highest capacity for glucose uptake in tumors, exceeding that of cancer cells and T cells on a per-cell basis^27^. Interestingly, 8 Gy of irradiation reduced MERTK^+^ macrophages by 50% (Extended Data Fig. 3a,b). As CAR T cell transcriptional signatures showed enhanced metabolic fitness (Extended Data Fig. 2a,c) and macrophages have been attributed with immune dysfunction in NSCLC^17,28^, we hypothesized that irradiation-induced depletion of these primary metabolic competitors and immune regulators may relieve nutrient competition and contribute to the enhanced metabolic activity and persistence of CAR T cells in irradiated tumors.

To test whether targeted depletion of macrophages alone could enhance CAR T cell persistence, we administered either anti-CSF1R^29,30^ or isotype antibody before adoptive transfer of syngeneic CAR T cells to mice that had established lung tumors (Extended Data Fig. 3c). Surprisingly, anti-CSF1R treatment had no measurable effect on survival of mice treated with CAR T cells (Extended Data Fig. 3d). Furthermore, anti-CSF1R treatment did not enhance CAR T cell cytotoxicity (Extended Data Fig. 3e) or persistence (Extended Data Fig. 3f,g). However, anti-CSF1R treatment was sufficient to abrogate upregulation of PD1 and TIGIT on CAR T cells (Extended Data Fig. 3h), suggesting that TRT may mitigate the upregulation of checkpoint and coinhibitory markers on CAR T cells in part through depletion of tumor-associated macrophages.

These data demonstrate that elimination of a dominant glucose-consuming population in the TME is insufficient to enhance CAR T cell therapy and the enhanced activity observed in CAR T cells from irradiated tumors is not attributable to depletion of these macrophages. We then pivoted to assess whether other APCs in the TME were regulating CAR T cells in irradiated tumors.

### DCs are required for CAR T cells persistence in irradiated tumors

**Extended Data Fig. 4:**
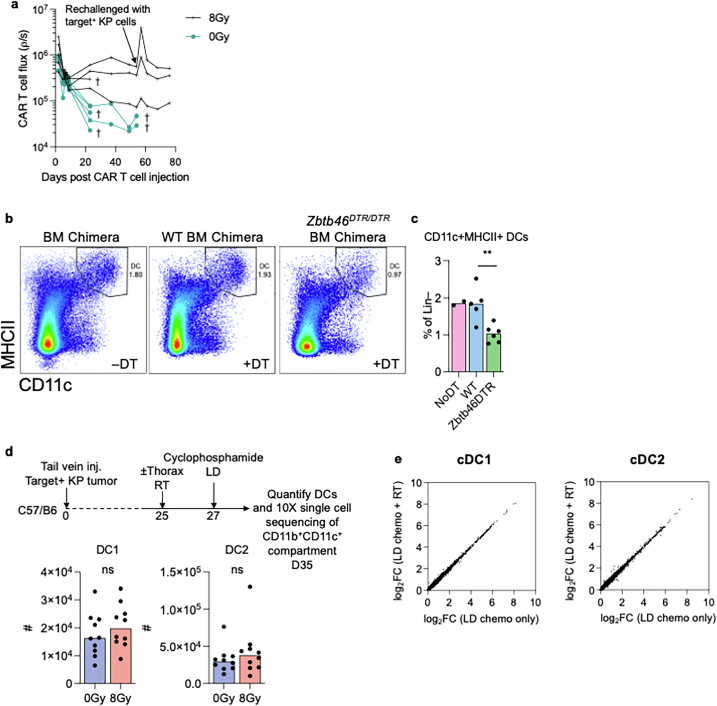
Quantification and characterization of changes in DCs in lung tumors with irradiation. **a**, Kinetics of thoracic CAR T cell persistence by Bioluminescence imaging. Mice inoculated with KP19 lung tumors were randomized to treatment with thorax radiotherapy as in Fig. 1a, and then lymphodepleted and treated with CBR Luciferase and CAR double transduced syngeneic mouse T cells. Lines track BLI from individual mice. Arrow indicates time point when mice were rechallenged with 5×10^5^ target^+^ KP19 cells intravenously. n = 5. Data representative of two independent experiments. In total, 9 surviving mice we rechallenged with tumor showing an average 2-fold increase in CAR T cell thoracic flux two-days after rechallenge. **b-c**, Representative flow cytometry plots and quantification (**c**) of single-cell suspensions from spleens of *Zbtb46*^*DTR/DTR*^ or WT BM chimeric mice (as in Fig. 3b) were stained for MHCII^+^ CD11c^+^ CD45^+^Lineage-negative (CD19^*−*^ CD3e^*−*^ NK1.1^*−*^ B220^*−*^) DCs. n = 5 (WT) n = 6 (Zbtb46-DTR) mice per DT treated group (n = 2 in no DT group shown for reference), two-sided t-test p value 0.0049 indicated by ** between groups indicated by line on plot. **d-e**, Mice inoculated with 5×10^5^ target^+^ CBR-Luciferase^+^ orthotopic KP lung tumors via tail vein-injection were distributed based on thoracic tumor luminescence to ensure equal distribution of thoracic tumor burden to receive either 8 Gy or no thoracic irradiation 25 days after tail-vein injections. They were then lymphodepleted and harvested on day 35-post tail-vein injections for quantification of dendritic cell populations (**d**; n = 10 mice per group) or for single cell sequencing of the sorted CD45^+^CD3^*−*^ mCD19^*−*^, Ly6G^*−*^, CD11b ^+^ CD11c ^+^ myeloid cell compartment (**e**; n = 3 mice per group.). DCs were defined as CD11b ^*−*^ Xcr1 ^+^ DC1s or CD11b ^+^ Xcr1 ^*−*^ DC2s within the following population: Live CD45^+^, Ly6G^*−*^, MERTK^*−*^, B220^*−*^, MHCII^+^ CD11c ^+^. **e**, Expression profiles from single cell sequencing of cDC1 and cDC2 sorted from mice treated with lymphodepleting chemotherapy alone or with 8 Gy of thoracic radiotherapy. Mice were harvested 10 days after radiotherapy for single cell isolation and sorting. Plots show Log_2_FC of gene expression. Log_2_ ratios were ~1 for all DEGs among cDC1 cells (left) and cDC2 cells (right), indicating similar transcriptome programs n = 3 mice per group. RT, radiotherapy, LD, lymphodepletion. Source data

CAR T cell bioluminescence remained elevated in irradiated mice for 2 months after adoptive cell transfer and increased after mice were rechallenged with target^+^ KP cells (Extended Data Fig. 4a). To expand and persist, T cells require strong proliferation signals from APCs, the most potent of which are DCs^31^. Furthermore, CD8^+^ CAR T cells in irradiated tumors were significantly enriched for expression of *Ccl5*, which encodes a cytokine involved in the recruitment of DCs to the TME^32^ (Fig. 2a). Therefore, we hypothesized that DCs were driving the enhanced persistence of CAR T cells in irradiated tumors.

**Fig. 3:**
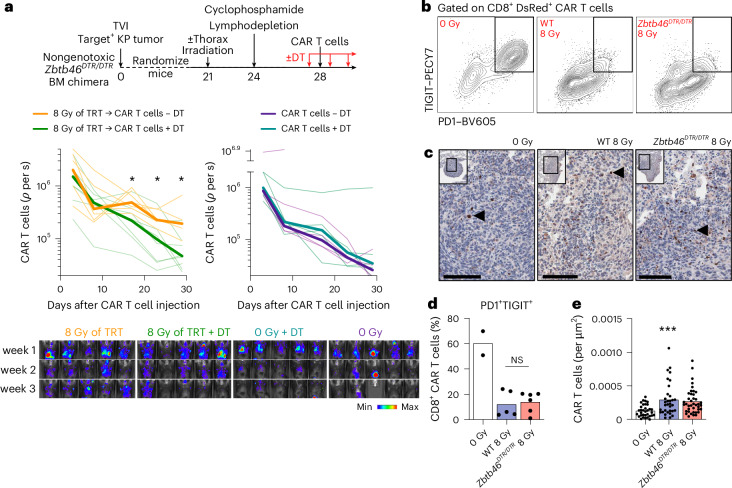
DCs are required for enhanced persistence of CAR T cells in irradiated tumors. **a**, *Zbtb46*^*DTR*/*DTR*^ nongenotoxic BM chimeras inoculated with 7 × 10^5^ target^+^ orthotopic KP lung tumors through TVI were randomized 3 weeks later to 8 Gy of TRT or no irradiation. Cohorts were then lymphodepleted and treated with Luciferase and CAR double-transduced syngeneic mouse T cells to a dose of 2.5 × 10^6^ CAR T cells per mouse. To ensure sustained depletion of DCs, DT was administered starting the day before CAR T cell injection and every 2–3 days after for the remainder of the experiment in the cohorts indicated. Kinetics of thoracic CAR T cell persistence by BLI were determined. Faint lines track bioluminescence from individual mice. Median values are shown in bold (*n* = 6 for no DT + 8 Gy, *n* = 7 for DT + 8 Gy and *n* = 5 for 0 Gy). Day 17, *P* = 0.0140; day 23, *P* = 0.0426; day 28, *P* = 0.0182, Mann–Whitney *U*-test. **b**,**d**, Representative flow cytometry plots (**b**) and quantification (**d**) of DsRed^+^ CAR T cells in the lungs of target^+^ KP tumor-bearing mice isolated 9 days after adoptive cell transfer and stained for expression of indicated surface markers. *Zbtb46*^*DTR/DTR*^ or WT BM chimeras were inoculated with 7 × 10^5^ target^+^ orthotopic KP lung tumors through TVI then treated 3 weeks later with 8 Gy of TRT. Cohorts were then lymphodepleted and treated with syngeneic T cells transduced with a CAR-2A–DsRed retroviral construct to a dose of 2.5 × 10^6^ CAR T cells per mouse. To ensure sustained depletion of DCs, DT was administered starting the day before CAR T cell injection and every 2–3 days after to both groups of mice (*n* = 5 for WT + 8 Gy, *n* = 6 for *Zbtb46*^*DTR/DTR*^ + 8 Gy). Statistical analysis conducted using a two-sided *t*-test. **c**, Representative paraffin sections of target^+^ KP tumor-bearing lungs isolated 9 days after CAR T cell infusion, stained for DsRed expression on CAR T cells (example indicated by black arrowhead; hematoxylin counterstained). Scale bars, 100 µm. **e**, Quantification of intratumoral DsRed^+^ CAR T cell density 9 days after infusion of CAR T cells. Each dot represents a tumor nodule from representative lung sections from representative lung sections from *n* = 5 (WT + 8 Gy, 33 tumors) or *n* = 6 (*Zbtb46*^*DTR/DTR*^ + 8 Gy, 40 tumors) mice per irradiated group (*n* = 4–10 tumors per lung section). Bars illustrate mean values. ***P = 0.0007, ordinary one-way ANOVA. Source data

Classical DCs, comprising two distinct subsets (DC1 and DC2)^33,34^, can be distinguished from other immune lineages by expression of the transcription factor *Zbtb46* (refs. ^35,36^). To time depletion of DCs, we used a knock-in mouse model in which an IRES–diphtheria toxin receptor (DTR)–mCherry cassette was knocked into the 3′-UTR of the *Zbtb46* locus^36^. The expression of *Zbtb46* on endothelial cells^35^ necessitates the generation of *Zbtb46*^*DTR*/*DTR*^ bone marrow (BM) chimeras in which DT administration does not deplete host endothelial cells or cause lethality^36^ but depletes DCs^36^ (Extended Data Fig. 4b,c). We used nongenotoxic depletion of host hematopoietic stem and progenitor cells with anti-CD117-conjugated saporin toxin^37^ to generate *Zbtb46*^*DTR/DTR*^ BM chimeras that were then injected with target^+^ KP cells and randomized for CAR T cell treatment (Fig. 3a). Remarkably, sustained depletion of DCs abolished the enhanced persistence of CAR T cells seen in irradiated mice. By contrast, CAR T cells in unirradiated mice declined with indistinguishable kinetics between DC depleted and nondepleted conditions (Fig. 3a). These data suggest that CAR T cells do not form productive interactions with DCs in unirradiated tumors but do so in irradiated tumors.

In addition, CD8^+^ CAR T cells maintained low levels of PD1 and TIGIT in irradiated tumors whether DCs were depleted or not (Fig. 3b,d). Furthermore, depletion of DCs did not impact early CAR T cell infiltration into tumors (day 9 after injection; Fig. 3c,e). Together, these data suggest that DCs were not driving enhanced infiltration of CAR T cells into irradiated tumors but were required for sustaining CAR T cells over the following weeks.

### Antigen-dressed DCs expand CAR T cells through the chimeric receptor

**Fig. 4:**
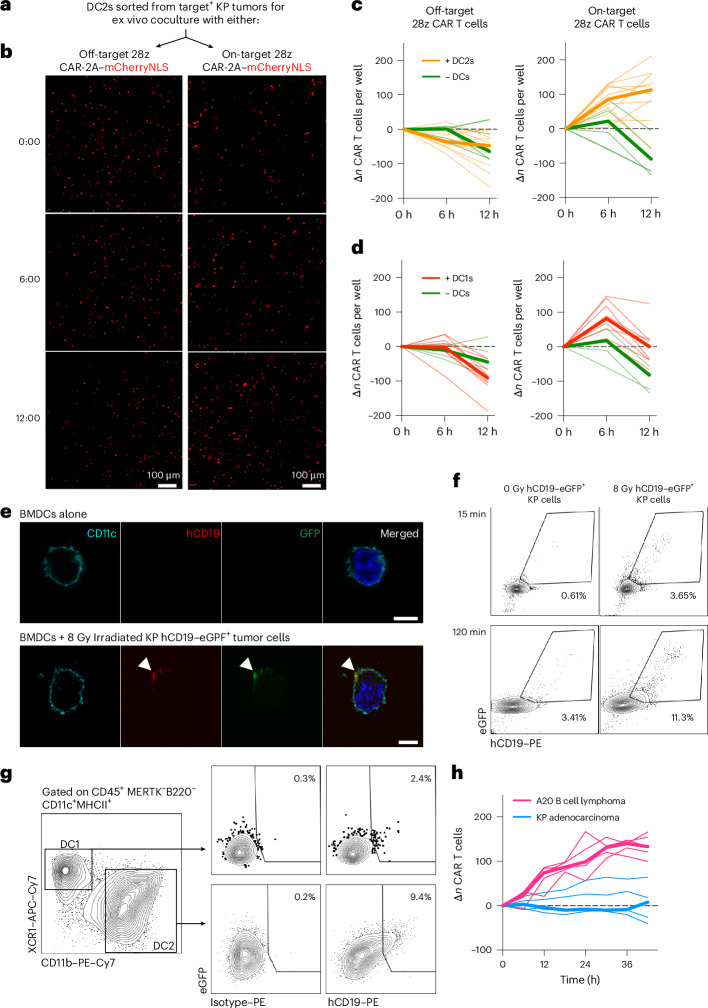
Antigen-dressed DCs activate CAR T cells through the chimeric receptor. **a**–**d**, Serial imaging (**a**) and quantification of CAR-2A-mCherryNLS transduced syngeneic T cells in cocultures with DC1 (**d**) or DC2 (**b**,**c**) sorted from target^+^ KP tumor-bearing lungs or with DC medium alone. On-target (h1928ζ-2A-mCherryNLS) CAR T cells, but not off-target (m1928ζ-2A-mCherryNLS) CAR T cells, have the scFv-binding domain that binds hCD19 antigen expressed by target^+^ KP tumors. **b**, Representative ×10 widefield fluorescence images of mCherryNLS^+^ CAR T cells at the indicated time points after addition of CAR T cells to CD11b^+^XCR1^*−*^ DC2s. Data representative of two independent experiments. Cells were cultured in DC medium supplemented with FLT3L. **c**,**d**, CAR T cell numbers counted over four ×10 widefield fluorescence images taken per replicate well at successive time points after addition of off-target (left) or on-target (right) CAR T cells to DC2 (**c**) or DC1s (**d**). Each faint line represents the change in number of CAR T cells per replicate well from baseline at *t* = 0 (*n* = 5 technical replicates per mouse). DCs sorted from two mice. Median values are in bold. Data representative of three independent experiments. **e**, BMDCs unfed at steady state or cocultured with hCD19–eGFP^+^ KP cells irradiated with 8 Gy for 1 h were stained with anti-CD11c, anti-GFP and anti-hCD19. Scale bars, 5 μm. Data representative of 14 images of BMDCs, showing surface colocalization of transferred tumor antigen with CD11c on BMDCs derived from one mouse BM. **f**, Representative flow cytometry plots gated on CD45^+^MHCII^+^CD11c^+^ BMDCs cocultured for 15 or 120 min with nonenzymatically dissociated hCD19–eGFP fusion-expressing KP cell lines that were irradiated or not (24 h before) and stained for surface positivity of hCD19–eGFP fusion protein with anti-hCD19. Data representative of two independent experiments (two BM donor mice per BMDC preparation for each of two independent experiments). **g**, Representative flow cytometry plots of DC1 and DC2 isolated from hCD19–eGFP^+^ KP tumor-bearing mice stained for surface positivity of hCD19–eGFP fusion protein with anti-hCD19 or isotype antibody. hCD19–eGFP^+^ KP cells were injected via TVI and allowed to grow for 4 weeks before isolation. Gating strategy shown in Extended Data Fig. 5h. Data representative of *n* = 4 tumor-bearing mice. **h**, h1928ζ-2A-mCherryNLS CAR T cell numbers counted over four ×10 widefield fluorescence images taken per replicate well at successive time points following addition to wells in a 1:1 E:T ratio with hCD19^+^ A20 B cell lymphoma or KP adenocarcinoma. BALB/c-derived CAR T cells were used in A20 cocultures; C57BL/6 cells were used in KP cocultures. Each faint line represents the change in number of CAR T cells per replicate well from baseline at *t* = 0 (*n* = 5 technical replicates). Data representative of two independent experiments. Source data

**Extended Data Fig. 5:**
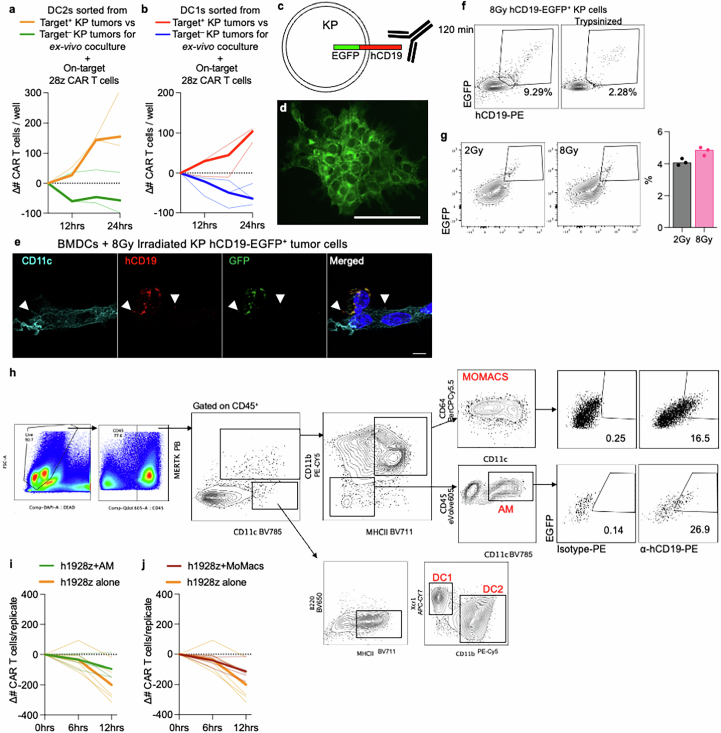
DCs sorted from target^+^ KP tumors and not target^−^ KP tumor-bearing lungs can expand CAR T cells ex vivo. **a-b**, Quantification of numbers of h1928z- transduced syngeneic T cells in serially-imaged cocultures with DC2 (**a**) or DC1 (**b**) sorted from target^+^ vs. target^*−*^ KP tumor-bearing lungs. On-target (h1928z-2A-mCherryNLS) CAR T cells have the SJ25C1 scFv binding domain that binds the target protein hCD19 expressed by target^+^ KP tumors, but not target^*−*^ KP tumor. 3 technical replicates per group shown. DCs sorted from single cell suspensions pooled from n = 2 mice per group. **c**, KP tumors were transduced with lentiviral constructs expressing a human CD19-EGFP fusion molecule that allowed for detection of cell-surface vs. intracellular localization of the molecule by surface staining of cells with α-hCD19 antibody and co-expression of EGFP. **d**, Fluorescence imaging of hCD19-EGFP^+^ KP clonal cell line. Fluorescence image representative of multiple routine imaging of these cell lines performed during passaging. Scale bar, 100um. **e**, BMDC co-cultured with 8Gy-irradiated hCD19-EGFP^+^ KP cells for 1 hour were fixed on glass coverslips and stained with anti-CD11c, anti-GFP and anti-hCD19 antibodies. Arrowheads indicate antigen-dressed hCD19-EGFP on CD11c+ cells. Bars: 5 μm. Representative of 14 total images of BMDCs. Bars: 5 μm. **f**, Representative flow cytometry plots gated on MHCII^+^CD11c^+^ bone marrow derived DCs (BMDC) cocultured for 2 hours with non-enzymatically dissociated 8Gy-irradiated hCD19-EGFP+ KP cells. For the trypsinization condition, non-enzymatically dissociated tumor cells were pelleted and resuspended in trypsin 0.025% solution for 5 minutes then neutralized with FBS-media prior to coculture. Two bone marrow donor mice per BMDC preparation for two independent experiment (second independent experiment shown in **g**). **g**, Representative flow cytometry plots gated on MHCII^+^CD11c^+^ BMDC cocultured for 2 hours with non-enzymatically dissociated 2 Gy vs 8Gy-irradiated hCD19-EGFP fusion-expressing KP cells. Right panel: Quantification of technical replicates showing percentage of BMDCs positive for hCD19-EGFP. n = 3 technical replicates per group. **h**, Gating strategy for Fig. 4g and representative flow cytometry plots of Monocyte-derived macrophages (MOMACS) and alveolar macrophages (AM) in cell suspensions isolated from hCD19-EGFP^+^ KP tumor-bearing mice, stained for surface positivity of hCD19-EGFP fusion protein with α-hCD19 or isotype antibody. hCD19-EGFP KP cells were injected into mice via the tail-vein and allowed to grow for 4-weeks prior to harvest. **i-j**, Quantification of numbers of h1928z-2A-mCherryNLS^+^ syngeneic T cells in serially-imaged cocultures with alveolar macrophages (**g**) or monocyte-derived macrophages (**h**) sorted from target^+^ KP tumor-bearing lungs. n = 5 technical replicates. Data representative from two independent experiments. Source data

Interestingly, 8 Gy of irradiation in the setting of lymphodepleting chemotherapy did not drastically alter the numbers (Extended Data Fig. 4d) or transcriptional programs of DCs repopulating tumor-bearing lungs (Extended Data Fig. 4e). To assess DC and CAR T cell interactions directly, syngeneic T cells transduced with CAR-2A-mCherryNLS^+^ constructs were added to culture wells that contained DC1 or DC2 freshly sorted from KP tumor-bearing lungs and the number of mCherryNLS^+^ CAR T cells was quantified on fluorescence images taken at serial time points thereafter (Fig. 4a,b). DCs sorted from target^+^ KP tumor-bearing lungs had the capacity to expand CAR T cells in coculture, while DCs sorted from target^*−*^ KP tumor-bearing lungs did not (Extended Data Fig. 5a,b). Therefore, we hypothesized that target antigens on DCs were activating CAR T cells through the chimeric receptor.

To test the requirement of antigen recognition by the chimeric receptor, we cocultured freshly sorted DC1s and DC2s from target^+^ KP tumor-bearing lungs with syngeneic T cells transduced with either an ‘on-target’ CAR, containing an scFv recognition domain that binds the target antigen, or an ‘off-target’ scFv that did not (Fig. 4a,b). The addition of DCs was not sufficient to increase CAR T cell numbers in the absence of target antigen recognition (off-target scFv) but did increase numbers of on-target CAR T cells (Fig. 4b–d and Extended Data Fig. 5a,b). Expansion of on-target CAR T cells was not due to endogenous TCR recognition of peptide-MHC presented on DCs, as off-target CAR T cells derived from the same spleen donor have identical TCR repertoires. Therefore, these data suggested that DCs were activating T cells through the chimeric receptor with CAR target antigens obtained from tumor cells.

While transfer of cell-surface proteins between immune cells has been reported in several contexts^38^, how cell-surface proteins are taken up and processed by DCs and the consequences of this for CAR T cell therapy have not been established. To determine whether DCs could obtain surface proteins directly from tumor cells, a process we refer to as ‘antigen dressing’, we cocultured BM-derived DCs (BMDCs) with nonenzymatically dissociated KP cells expressing human CD19 with eGFP fused to the intracellular domain (Extended Data Fig. 5c,d). Confocal microscopy of cocultured BMDCs visualized hCD19 and eGFP staining colocalized with CD11c on the cell membrane (Fig. 4e and Extended Data Fig. 5e). BMDCs underwent antigen dressing from irradiated hCD19–eGFP^+^ KP cells after only 15 min of coculture. Tumor cell irradiation increased antigen dressing of hCD19–eGFP onto BMDCs after 2 h of coculture (Fig. 4f). Interestingly, trypsinization of irradiated tumor cells before coculture abrogated antigen dressing (Extended Data Fig. 5f), suggesting that trypsin-sensitive moieties on tumor cells provide substrates for transfer of antigen to BMDCs. BMDC antigen dressing of hCD19–eGFP^+^ KP cells irradiated with a 2-Gy dose was comparable to tumor cells irradiated with an 8-Gy dose (Extended Data Fig. 5g). Together, these data suggest that antigen dressing can occur through trogocytosis of surface proteins within minutes of conjugate formation between live cells, in contrast to phagocytosis and internalization that takes place over hours^38^.

To detect DC antigen dressing in vivo, we injected mice with hCD19–eGFP^+^ KP cells (Extended Data Fig. 5c,d). Surface staining of live singlets with anti-hCD19 antibody revealed a clear shift in eGFP-positive DCs compared to isotype-stained samples (Fig. 4g), demonstrating that DCs undergo antigen dressing of tumor-derived surface antigens in vivo.

We also observed antigen dressing onto macrophages (Extended Data Fig. 5h). Although macrophages are also professional APCs, they failed to expand mCherryNLS^+^ CAR T cells in ex vivo cocultures (Extended Data Fig. 5i,j). This finding suggests that the identity of the target cell dictates the fate of the CAR T cell effector, leading us to question whether other professional APCs, namely B cell lineages, could expand CAR T cells. Syngeneic mCherryNLS^+^ CAR T cells expanded when targeted to hCD19^+^ A20 B cell lymphomas but not hCD19^+^ KP adenocarcinomas (Fig. 4h), suggesting that B cell lineages also harbor additional stimulatory signals that can expand CAR T cells. These data may explain in part the durable persistence of B cell-targeted CAR T cells in the absence of tumor irradiation.

### Sustained CAR T cell activity required for durable control of irradiated tumors

**Fig. 5:**
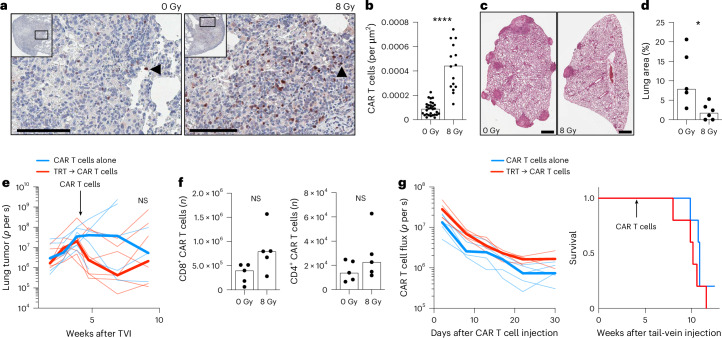
Sustained CAR T cell activity required for durable control of irradiated tumors. **a**–**e**, *Batf3*^−/−^ mice inoculated with 5 × 10^5^ target^+^ orthotopic KP lung tumors through TVI were randomized 3 weeks later to 8 Gy of TRT or no irradiation. Cohorts were then lymphodepleted and treated with syngeneic T cells transduced with a CAR-2A–DsRed retroviral construct to a dose of 4 × 10^6^ CAR T cells per mouse. **a**, Representative paraffin sections of tumor-bearing lungs stained for DsRed in adoptively transferred CAR T cells (selected example indicated by black arrowhead; hematoxylin counterstain). Tumor-bearing lungs were isolated 9 days after CAR T cell transfer for histologic analysis. Scale bar, 100 µm. **b**, Quantification of intratumoral DsRed^+^ CAR T cell density. Each dot represents a tumor nodule from representative lung sections from *n* = 5 (0 Gy, 30 tumors) or *n* = 6 (8 Gy, 16 tumors) mice (*n* = 4–10 tumors per lung section). ****P < 0.0001, Mann–Whitney *U*-test. **c**,**d**, Representative H&E images (**c**) and quantification (**d**) of tumor-bearing lungs of mice on day 9 after CAR T cell therapy. Quantification of the tumor area as a percentage of the total area of the lung cross-section (right) is shown (*n* = 5 for 0 Gy, *n* = 6 for 8 Gy). *P = 0.0177, two-sided *t*-test. Scale bar, 1 mm. **e**, Kinetics of Luciferase^+^ KP19 thoracic tumor bioluminescence. Male mice were randomly allocated to 8 Gy of TRT or no irradiation treatment groups on the basis of thoracic tumor luminescence at 2 weeks following TVI of tumor cells as in **a** and then lymphodepleted and treated with CAR-transduced WT male C57BL/6 T cells. Faint lines track tumor bioluminescence from individual mice. Median values are shown in bold (*n* = 5 for 0 Gy, *n* = 7 for 8 Gy). Statistical analysis conducted using a two-sided *t*-test. **f**, Quantification of DsRed^+^ CAR T cells in the lungs of KP19 tumor-bearing mice isolated 9 days after adoptive transfer of CAR T cells (*n* = 5 mice). Statistical analysis conducted using a two-sided *t*-test. **g**, Kinetics of CAR T cell persistence by thoracic bioluminescence (left) and survival (right) in *Batf3*^−/−^ mice. *Batf3*^−/−^ mice inoculated with 5 × 10^5^ target^+^ orthotopic KP lung tumors through TVI were randomized 3 weeks later to TRT or no irradiation. Cohorts were then lymphodepleted and treated with Luciferase and CAR double-transduced syngeneic mouse T cells to a dose of 2.5 × 10^6^ CAR T cells per mouse (*n* = 5). Faint lines track CAR T cell bioluminescence from individual female mice. Median values are shown in bold. Results are representative of two experiments reproduced in male and female cohorts. Source data

Antigen dressing was detectable on DC1 (Fig. 4g) that expanded CAR T cells to a lesser degree than DC2 (Fig. 4c,d and Extended Data Fig. 5a,b). However, DC1 has a critical role for cytotoxic T cell immunity^39–41^. Therefore, we tested whether irradiation would enhance CAR T cell efficacy in mice deficient for the transcription factor *Batf3* and lack DC1s^39^. Once again, the numbers of tumor-infiltrating DsRed^+^ CAR T cells were increased (Fig. 5a,b) and tumor burden was reduced in irradiated *Batf3*^−/−^ mice (Fig. 5c,d). However, bioluminescence imaging (BLI) of thoracic tumor revealed that this reduction was not sustained (Fig. 5e). Critically, irradiation did not affect CAR T cell persistence or survival of irradiated *Batf3*^−/−^ mice (Fig. 5f,g). These data demonstrate that sensitization of tumors to cytotoxicity by irradiation is not sufficient to control solid tumors and highlight the requirement for sustained activity of CAR T cells.

### Irradiation enhances CAR T cell expansion in tumors but not adjacent normal tissue

**Fig. 6:**
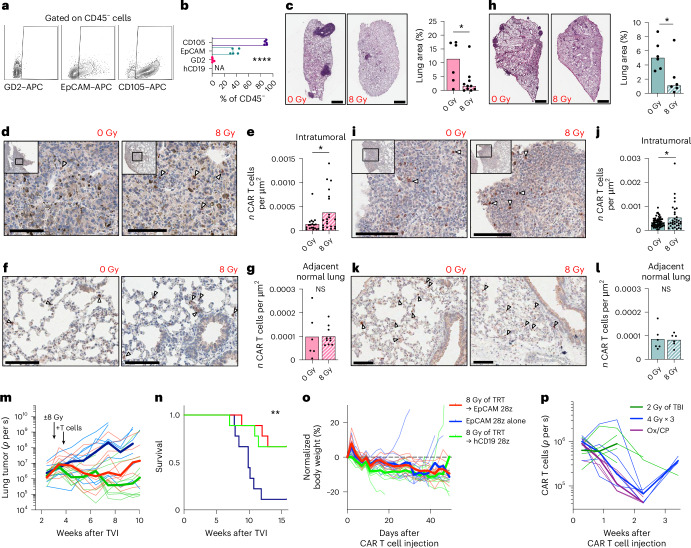
Robust efficacy of CAR T cells targeted to endogenous antigens on irradiated tumors. **a**,**b**, Representative flow cytometry plots (**a**) and quantification (**b**) of single-cell suspension of non-tumor bearing WT lungs stained for markers indicated (*n* = 5). ****P < 0.0001, ordinary one-way ANOVA. **c**–**g**, C57BL/6 mice inoculated with 1 × 10^5^ B16-F10 melanoma cells through TVI were randomized 3 weeks later to 8 Gy of TRT or no irradiation. Cohorts were then lymphodepleted and treated with syngeneic T cells transduced with GD2 CAR-2A–DsRed retroviral construct to a dose of 3 × 10^6^ CAR T cells per mouse and lungs were isolated 9 days after CAR T cell transfer. **c**, Representative H&E sections of tumor-bearing lungs. Right, quantification of the tumor area as a percentage of the total area of the lung cross-section. Each dot represents a mouse (*n* = 9–10 mice per group at time of CAR T cell injection, but only *n* = 6 mice in the unirradiated group survived until the time of tissue isolation; *n* = 10 for 8 Gy). *P = 0.0302, two-sided *t*-test. Scale bars, 1 mm. **d**,**e**, Representative paraffin sections (**d**) and quantification (**e**) of B16-F10 melanoma-bearing lungs stained for DsRed expression on GD2 CAR T cells (dots represent density in tumor nodules from representative lung sections; *n* = 4–10 tumors per lung section; *n* = 6 for 0 Gy (19 tumors), *n* = 10 for 8 Gy (21 tumors)). *P = 0.0227, two-sided *t*-test. **f**,**g**, Representative paraffin sections (**f**) and quantification (**g**) of DsRed^+^ GD2 CAR T cells in uninvolved lung parenchyma of B16-F10 melanoma-bearing lungs (dots represent cell numbers per nontumorous sample area per section; *n* = 6 for 0 Gy, *n* = 10 for 8 Gy). Statistical analysis conducted using a two-sided *t*-test. DsRed expression on GD2 CAR T cells is shown (hematoxylin counterstain). Arrowheads indicate DsRed^+^ CAR T cells. Scale bars, 100 µm. **h**–**l**, C57BL/6 mice inoculated with 5 × 10^5^ KP lung tumors through TVI were randomized 3 weeks later to 8 Gy of TRT or no irradiation. Cohorts were then lymphodepleted and treated with syngeneic T cells transduced with EpCAM CAR-2A–DsRed retroviral construct to a dose of 3 × 10^6^ CAR T cells per mouse and lungs were isolated 9 days after CAR T cell transfer. **h**, Left: representative H&E sections of tumor-bearing lungs of mice on day 9 after CAR T cell therapy. Scale bars, 1 mm. Right: quantification of tumor area as a percentage of the total area of the lung cross-section. Each dot represents a mouse (*n* = 6). *P = 0.0450, two-sided *t*-test. **i**,**j**, Representative paraffin sections (**i**) and quantification (**j**) of KP tumor-bearing lungs stained for DsRed expression on EpCAM CAR T cells (dots represent density in tumor nodules from representative lung sections from *n* = 6 mice per group (0 Gy, 63 tumors; 8 Gy, 33 tumors; *n* = 4–10 tumors per lung section)). *P = 0.0131, two-sided *t*-test. **k**,**l**, Representative paraffin sections (**k**) and quantification (**l**) of DsRed^+^ EpCAM CAR T cells in uninvolved lung parenchyma in KP tumor-bearing lungs (dots represent cell numbers per nontumorous sample area per section; *n* = 6 mice). Statistical analysis conducted using a two-sided *t*-test. DsRed expression on EpCAM CAR T cells is shown (hematoxylin counterstain). Arrowheads indicate DsRed^+^ CAR T cells. Scale bars, 100 µm. **m**–**o**, hCD19^+^EpCAM^+^ Luciferase^+^ KP lung-tumor-bearing mice were randomized to irradiation or left unirradiated and then randomized to treatment with 3 × 10^6^ CAR T cells targeted to either hCD19 or to EpCAM. Thoracic tumor bioluminescence kinetics (**m**), survival (**n**) and normalized body weight (**o**) after treatment with CAR T cells as indicated (*n* = 9 for 0 Gy, *n* = 10 for 8 Gy). Faint lines track values from individual mice. Median values shown in bold. **P = 0.0028, log-rank (Mantel–Cox) test. **p**, Kinetics of EpCAM CAR T cell persistence by thoracic bioluminescence in C57BL/6 mice inoculated with 5 × 10^5^ orthotopic KP lung tumors through TVI and then randomized 3 weeks later to indicated treatment. The oxaliplatin/cyclophosphamide (Ox/CP) group was treated with 2.5 mg kg^−1^ oxaliplatin and 150 mg kg^−1^ cyclophosphamide 3 days before CAR T cells. Irradiation was delivered 7 days before CAR T cells. Cohorts were then treated with Luciferase and CAR double-transduced syngeneic mouse T cells to a dose of 2.5 × 10^6^ EpCAM CAR T cells per mouse (*n* = 5 per group). Faint lines track CAR T cell bioluminescence from individual mice. Median values shown in bold. Source data

**Extended Data Fig. 6:**
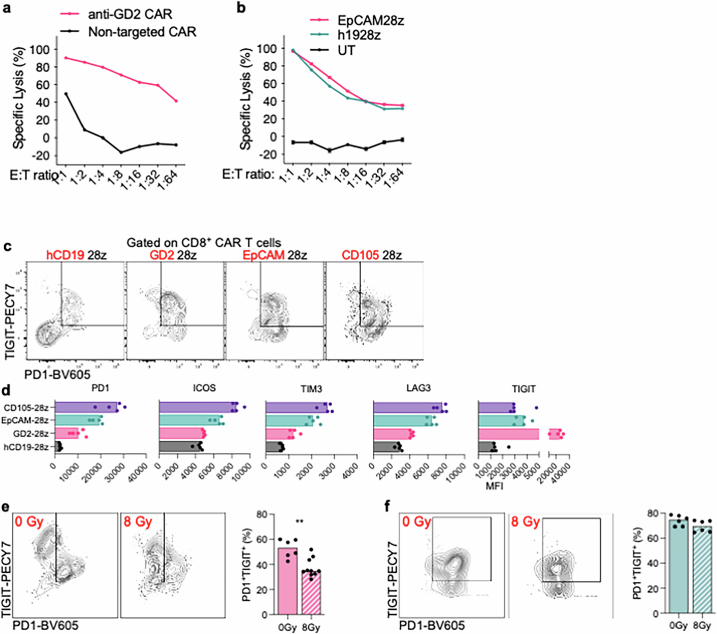
Generating CAR T cells to endogenously expressed tumor antigens. **a**, 2.5×10^4^ Luciferase^+^ B16-F10 melanoma cells were added to coculture at indicated effector-to-target (E:T) ratios with GD2-CAR T cells and cultured for 18 hours. Mean ± SEM, 12 replicates per dilution. **b**, 5×10^4^ hCD19^+^ Luciferase^+^ KP cells were added to coculture at indicated E:T ratios with either hCD19- or EpCAM-targeted CAR T cells at the indicated E:T ratios and cultured for 18 hours. Mean ± SEM, 12 replicates per dilution. **c-d**, Representative flow cytometry plots and MFI (**d**) of indicated markers on CAR T cells in the lungs of WT mice harvested 4 days after adoptive transfer of 2.5×10^6^ CAR T cells per mouse. CAR-2A-DsRed retroviral constructs differed only in the sequence encoding for the scFv recognition domains indicated in red; n = 5. **e**, C57BL/6 mice inoculated with 1×10^5^ B16-F10 melanoma cells via tail vein-injection were randomized three weeks later to 8 Gy thoracic irradiation or no irradiation. Cohorts were then lymphodepleted and treated with syngeneic T cells transduced with GD2 CAR-2A-DsRed retroviral construct to a dose of 3×10^6^ CAR T cells per mouse, and lungs were harvested 9-days after CAR T cell transfer. Representative cytometry plots and quantification (right panel) of indicated surface markers on CD8^+^ GD2 CAR T cells 9-days post adoptive transfer. n = 6 (0 Gy) n = 10 (8 Gy) mice per group. Two-tailed t-test ** p value 0.0013. **f**, C57BL/6 mice inoculated with 5×10^5^ KP lung tumors via tail vein-injection were randomized three weeks later to 8 Gy thoracic irradiation or no irradiation. Cohorts were then lymphodepleted and treated with syngeneic T cells transduced with EpCAM CAR-2A-DsRed retroviral construct to a dose of 3×10^6^ CAR T cells per mouse, and lungs were harvested 9-days after CAR T cell transfer. Representative cytometry plots and quantification (right panel) of indicated surface markers on CD8^+^ EpCAM CAR T cells 9-days post adoptive transfer. n = 6 mice per group. UT, untransduced. Source data

The enhanced expansion of CAR T cells in irradiated tumors raised the concern that irradiation might also increase CAR T cell numbers in normal tissues expressing the target antigen, thereby exacerbating on-target, off-tumor toxicity. To test this, we generated 28ζ-based mouse CAR constructs with an scFv specific to endogenously expressed tumor antigens (Extended Data Fig. 6a,b). We selected CAR target antigens with varying abundance in the stromal tissue of mouse lungs, ranging from a small fraction to the majority (Fig. 6a,b). The disialoganglioside GD2, expressed on melanoma, neuroblastoma and glioma^42,43^, was detectable on 1% of stromal cells (high tumor selectivity). By contrast, epithelial cell adhesion molecule (EpCAM), expressed highly on normal epithelia and epithelial-derived carcinomas^44,45^, was found on 37% of stromal cells (lower tumor selectivity).

Mice harboring advanced B16-F10 melanoma or KP adenocarcinoma lung metastases were randomized to TRT or left unirradiated and treated with GD2 CAR T cells (B16-F10 tumor-bearing mice; Fig. 6c–g and Extended Data Fig. 6e) or EpCAM CAR T cells (KP tumor-bearing mice; Fig. 6h–l and Extended Data Fig. 6f) and harvested 9 days later. As with hCD19 28ζ CAR T cells (Fig. 1), irradiation increased intratumoral CAR T cell numbers and reduced tumor burden when targeted to GD2 (Fig. 6c–e) or EpCAM-expressing tumors (Fig. 6h–j), demonstrating the robustness of these effects across distinct cancer histologies and antigen classes.

Notably, despite 37-fold more EpCAM^+^ than GD2^+^ cells in normal lung tissue (Fig. 6a,b), the numbers of GD2-targeted and EpCAM-targeted CAR T cells in normal lung parenchyma remained equivalently low in both irradiated and unirradiated mice (Fig. 6f,g,k,l). These data demonstrate that irradiation drives potent CAR T cell expansion selectively within the TME, even when the target antigen is abundantly present on adjacent healthy tissue.

#### Irradiation widens the therapeutic window of CAR T cell therapy

The differential expansion of CAR T cells in irradiated tumor suggested that irradiation could enhance efficacy without proportionally increasing toxicity. To test this directly, we compared hCD19 CAR or EpCAM CAR T cells in a head-to-head experiment. These CAR constructs had equivalent cytotoxicity against hCD19^+^EpCAM^+^ KP adenocarcinoma cells in vitro (Extended Data Fig. 6b). Because severe pulmonary toxicity was previously observed in mice treated with 10 × 10^6^ but not 3 × 10^6^ EpCAM CAR T cells^46^, we evaluated whether irradiation increased toxicity at this lower dose. Tumor-bearing mice were treated with either 8 Gy of TRT or left unirradiated before adoptive transfer of syngeneic hCD19 or EpCAM 28ζ CAR T cells.

Irradiated mice showed a pronounced and sustained reduction in tumor bioluminescence after treatment with EpCAM 28ζ CAR T cells (Fig. 6m), producing survival curves that overlapped with those of irradiated mice treated with hCD19 28ζ CAR T cells (Fig. 6n). However, EpCAM 28ζ CAR T cells had no antitumor effect in unirradiated mice. Critically, despite enhanced efficacy, irradiated mice treated with EpCAM 28ζ CAR T cells showed no increase in weight loss compared to unirradiated controls or to mice receiving the same dose of hCD19 28ζ CAR T cells (Fig. 6o) and no acute lethality was observed. These data demonstrate that irradiation widens the therapeutic window for CAR T cells targeted to endogenously expressed target antigens.

In non-tumor-bearing mice, CD8^+^ CAR T cells targeting endogenous antigens exhibited PD1 and coinhibitory receptor expression that scaled with antigen prevalence on lung stroma (Extended Data Fig. 6c,d). This correlation suggested that chronic antigen encounter in normal tissues might drive exhaustion and impair CAR T function against low-selectivity targets. For GD2, a highly selective tumor antigen in lung, irradiated mice showed reduced PD1^+^TIGIT^+^ staining compared to unirradiated mice (Extended Data Fig. 6e), mirroring the findings seen when the target antigen was hCD19 (Fig. 2g,h). Interestingly, EpCAM CAR T cells displayed elevated staining of these markers in irradiated KP tumor-bearing mice (Extended Data Fig. 6f), yet the strategy improved tumor control. Collectively, these findings demonstrate the robustness of this combination therapy in targeting antigens with varying levels of tumor selectivity.

To further contextualize our findings in clinically relevant settings, we tested whether three fractions of 4 Gy of TRT (totaling 12 Gy, with a biological effective dose (BED) of 28 Gy for *α*/*β* = 3, comparable to the BED of 29.3 Gy for a single fraction of 8 Gy) or 2 Gy of TBI could sustain CAR T cell levels. We also evaluated oxaliplatin and cyclophosphamide, an immunogenic chemotherapy regimen previously shown to enhance CAR T cell efficacy against lung tumors^47^, to determine its effect on CAR T cell persistence. Interestingly, three fractions of 4 Gy led to a delayed increased in EpCAM CAR T cell levels, whereas oxaliplatin and cyclophosphamide did not (Fig. 6p). These data indicate that focal high-dose irradiation regimens can enhance CAR T cell efficacy and additional optimization may adapt this strategy to various clinical scenarios.

## Discussion

Despite the transformative impact of immunotherapies, metastatic solid tumors remain the primary driver of cancer mortality worldwide^48,49^. Extensive disease is a major negative prognostic factor for response to both checkpoint blockade and cellular immunotherapy. Larger tumors impair immune responses^47^, with both preclinical and clinical studies demonstrating reduced effectiveness of PD1 blockade^50^ and cellular therapies against bulky disease^51,52^. Participants enrolled in CAR T cell trials for solid tumors typically have extensive disease and, to date, this promising technology has not achieved the same outcomes as CD19 CAR T cell therapy for B cell malignancies.

Here, we resolve how distinct effects of tumor irradiation overcome key barriers to CAR T cell therapy in orthotopic mouse models of extensive metastatic lung adenocarcinoma and melanoma. Our data support an updated radiobiological model in which irradiation acts through interconnected mechanisms. First, irradiation conditions tumor cells for antigen transfer to DCs (Fig. 4f and Extended Data Fig. 5f). Second, irradiation licenses productive DC–CAR T cell engagement: DC depletion impacted CAR T cell persistence only in irradiated tumors (Fig. 3a), indicating that this interaction is functionally absent without irradiation. Third, CAR T cells expanded selectively within irradiated tumors but not in adjacent normal tissue where the same antigen was widely expressed (Fig. 6f,g,k,l), suggesting that differential effects of irradiation on tumor versus normal tissue drive spatially restricted DC:CAR T cell organization. Critically, sustained CAR T cell persistence required an intact DC compartment. In its absence, tumors relapsed despite strong initial responses (Figs. 3a and 5e–g), underscoring that durable tumor control depends on DC-mediated immune conditioning.

Unexpectedly, DCs engaged CAR T cells through the chimeric receptor. We show that DCs acquire intact surface antigens from tumor cells through a trogocytic process of antigen dressing. As professional phagocytes, DCs express diverse families of receptors that recognize damaged and dead cells (adhesion receptors, TAM and scavenger receptors and C-type lectin receptors) that may contribute to antigen dressing and we do not exclude processes other than trogocytosis that may contribute to the display of native tumor antigen on DCs. Tumor irradiation facilitated engagement of antigen-dressed DCs with CAR T cells, as this interaction was not evident in unirradiated mice. Antigen-dressed DCs were found in tumor-bearing lungs, suggesting that these cells engage CAR T cells in the vicinity of lung tumors, in contrast to vaccination-based approaches that label DCs in the lymph nodes with target antigens and expand CAR T cells systemically^12,13^.

Interestingly, while B cell lymphoma could also expand CAR T cells, antigen-dressed macrophages did not, demonstrating that the nature of the target cell can impact the fate of the effector. This likely reflects an immunoregulatory bias of these macrophage subsets, as CAR T cells targeting F4/80^+^ macrophages are cleared from circulation within 10 days of infusion^53^. Moreover, these findings offer an additional explanation for the striking disparity in outcomes of CAR T cell therapy between B cell cancers and solid tumors and posit that the success of second-generation CARs against solid tumors may be contingent on harnessing DC-derived signals.

While further studies are needed to optimize this strategy for the clinic, our data provide important guideposts. Low doses of focal irradiation or TBI did not enhance CAR T cell persistence in our models of extensive lung metastases. Although prior work has shown that low-dose local radiotherapy^21^ or low-dose TBI^22^ can enhance CAR T cell efficacy against hematologic cancers in mouse models and although there is an active clinical trial investigating low-dose radiotherapy to bridge participants to CAR T cells for persons with large B cell lymphoma^54^, our data and prior work^55^ suggest that low-dose radiotherapy may not work in the context of histologies such as NSCLC or melanoma, which are amongst the most radioresistant^24^, in contrast to lymphoid lineages, which are radiosensitive^23^ (Extended Data Fig. 1j). These findings suggest that the capacity of radiation to facilitate DC–CAR T cell interactions is a distinct radiobiological endpoint. Hypofractionated conformal regimens may be ideally suited to achieve this in individuals. Whether radiotherapy parameters should be optimized for immune conditioning warrants prospective investigation. To irradiate the widely disseminated advanced lesions in our lung metastasis model, we used anterior–posterior fields to treat the bilateral lungs with doses not associated with radiation-induced pulmonary toxicity in mice or nonhuman primates^18^. However, recent data suggest that tumor-directed hypofractionated radiotherapy is sufficient to enhance immunotherapy for NSCLC^56,57^. Thus, while not specifically tested in our study, we speculate that conformal tumor-directed radiotherapy that spares uninvolved tissue can achieve the same effects reported here and mitigate risks of pulmonary toxicity.

The role of the endogenous T cell compartment in supporting a CAR T cell response has been reported in certain contexts in individuals^58–60^ and in preclinical models^21,61–63^ and it is interesting to speculate whether endogenous T cells contribute to CAR T cell treatment of irradiated tumors. However, we found that endogenous T cell numbers were decreased in both lungs and lymph nodes of irradiated mice compared to unirradiated controls (Fig. 1j and Extended Data Fig. 1i). Moreover, a comparison of survival curves from irradiated KP lung-tumor-bearing mice treated with nontargeted CAR T cells (Fig. 1d) versus those that received irradiation alone (Extended Data Fig. 1l) indicates that endogenous T cell responses did not significantly impact tumor control in this context and in this model, whether tumors expressed hCD19 or not.

Critically, a prospective randomized trial found that 12 Gy of TBI with autologous CD34^+^ hematopoietic stem cell rescue in addition to nonmyeloablative chemotherapy did not improve CR rates of TIL therapy for participants with metastatic melanoma^64^. As tumor-directed radiotherapy can spare the BM and minimize disruption of myelopoiesis, in contrast to systemic genotoxic agents or TBI, it may be better suited to maintain the conditions that generate the immune cascades for tumor clearance. Intratumoral DCs acutely decline by more than 80% after 4–12 Gy of irradiation and then normalize within 6 days^36,65^. Therefore, CAR T cells infused 7 days after irradiation encounter tumors with restored DC populations^18,56,57^ (Extended Data Fig. 4d,e).

Other pleiotropic effects of irradiation including altered tumor metabolic activity are not excluded. Doses of radiotherapy > 5 Gy reduce tumor uptake of tracer-labeled glucose 5–6 days after irradiation^66–69^. These data suggest that tumor metabolic activity is significantly reduced at the time that we administer CAR T cells. The upregulation of metabolic pathways in CAR T cells in irradiated tumors (Extended Data Fig. 2a,c) may reflect increased availability of glucose, fatty acids^70^ and other metabolites in irradiated tumors. Further study of the metabolic impact of irradiation on tumor may aid the effective clinical translation of this approach, for example, whether alternative hypofractionated regimens that achieve equivalent metabolic endpoints to a single 8Gy fraction might yield comparable therapeutic effects. Notably, recent work demonstrated that ultrahigh-dose-rate (>80 Gy s^−1^) radiotherapy enhances CAR T cell efficacy through macrophage-centered metabolic reprogramming^71^, although durable CAR T cell persistence was not observed. The sustained CAR T cell numbers seen here with conventional dose rates (2.1 Gy min^−1^) may reflect the inclusion of lymphodepleting chemotherapy in the conditioning regimen, as well as the distinct immune microenvironments of brain versus lung tumors.

The lack of persistence in *Batf3*^−/−^ mice despite equivalent irradiation highlights the primacy of DC-mediated immunology to the findings presented. However, DC-mediated activation of T cells increases their metabolic demands^72^ and the metabolic gene signatures observed in CAR T cells from irradiated tumors may, therefore, reflect the downstream consequences of sustained DC engagement rather than an independent effect of irradiation on nutrient availability. Consistent with this interpretation, depletion of macrophages, a dominant glucose consumer in the TME, was insufficient to enhance CAR T cell persistence (Extended Data Fig. 3). Instead, irradiation may create a metabolically permissive microenvironment that accommodates the heightened demands of DC-activated CAR T cells.

While our data establish that DCs are required for sustained CAR T cell persistence in irradiated tumors, the precise signals that DCs provide beyond antigen display remain to be defined. Second-generation CARs incorporate costimulatory domains, yet the failure of antigen-dressed macrophages to expand CAR T cells (Extended Data Fig. 5h,i) suggests that additional DC-specific signals are required. Several nonmutually exclusive possibilities include DC-specific costimulatory ligands or cytokines that integrate with CAR signaling to sustain proliferation. Moreover, the differential contributions of DC subsets merit consideration; while DC2 drove greater CAR T cell expansion ex vivo (Fig. 4c,d), DC1 was essential for durable persistence in vivo (Fig. 5f,g), suggesting that these subsets provide complementary signals. We also cannot exclude contributions from other cell types. For example, the enrichment of IL-2 signaling pathways observed in CAR T cells from irradiated tumors (Fig. 2b and Extended Data Fig. 2c) implicates this essential axis, potentially through autocrine or paracrine signaling. Future studies (CRISPR screening and RNA sequencing) may further elucidate DC-specific signals driving the effects reported here.

In conclusion, we demonstrate the robustness of this approach targeting both protein and ganglioside antigens with low or high selectivity for tumor. By locally amplifying CAR T cell effects without proportionally increasing toxicity in normal tissues, tumor irradiation may widen the therapeutic window, offering a path toward effective CAR T cell therapy for solid malignancies.

## Methods

All animal procedures were approved by the Institutional Animal Care and Use Committee (IACUC; protocol no. TR202500000026) of the Icahn School of Medicine at Mount Sinai. Mice within experiments were matched by age and sex. Irradiation experiments requiring randomization of mice were performed with female mice. All studies performed on mice were conducted in accordance with the IACUC at the Icahn School of Medicine at Mount Sinai.

### Mouse strains

Mice (6–12 weeks of age at the start of experiments) were used following a protocol approved by the Mount Sinai IACUC. Mice were maintained at specific-pathogen-free health status in individually ventilated cages at 21–22 °C and 39–50% humidity. C57BL/6J (000664), BALB/cJ (000651), B6(Cg)-*Zbtb46*^*tm1*(*HBEGF*)*Mnz*^/J (*Zbtb46*-DTR; 019506), CD45.1 C57BL/6J-*Ptprc*^*em6Lutzy*^/J (033076) and B6.129S(C)-*Batf3*^*tm1Kmm*^/J (013755) mice were purchased from The Jackson Laboratory. WT C57BL/6, CD45.1, *Zbtb46*^*DTR/DTR*^ and *Batf3*^−/−^ mice were either bred at the Icahn School of Medicine at Mount Sinai or purchased from The Jackson Laboratory and housed for a minimum of 7 days before experimental use.

### Cell lines and culture conditions

KP (from the M.M. lab) and A20 (American Type Culture Collection (ATCC), TIB-208) cell lines were transduced to express CAR target antigen human CD19 using vesicular stomatitis virus glycoprotein G (VSV-G) pseudotyped lentiviral supernatants derived from 293T cells transfected with self-inactivating lentiviral transfer plasmid (pLM backbone)^73^. Stably transduced cells were single-cell-sorted to generate clonal cell lines before use in mice. KP19 clonal lines and B16-F10 melanoma cells (ATCC, CRL-6475) were transduced with eGFP–CBR-Luciferase retroviral vectors to generate CBR-Luciferase^+^ KP19 or B16-F10 cells. Cells were tested for *Mycoplasma* using PCR of DNA extracts from lysed cell pellets (forward primer sequence, CGCCTGAGTAGTACGTTCGC; reverse primer sequence, GCGGTGTGTACAAGACCCGA). Gel electrophoresis was used to visualize which PCR products came from *Mycoplasma*-positive and *Mycoplasma*-negative cells. *Mycoplasma*-negative cells were expanded and batches were frozen for future use.

### Mouse tumor models

To model primary lung adenocarcinoma, mice were intravenously (i.v.) injected through the tail vein with KP engineered to express human CD19 (KP19), KP cells expressing both human CD19 and eGFP–CBR-Luciferase (CBR-Luciferase^+^ KP19) or KP cells expressing a human CD19–eGFP fusion protein (5 × 10^5^–7 × 10^5^ cells per mouse in 200 µl of PBS). KP cells were grown in complete cell culture medium (DMEM + 10% FBS + 1% penicillin–streptomycin + 2 mM glutamine) and were detached for use at 70% confluence using 0.25% trypsin. The tumor cells were originally derived from KP mice^74^ generated by crossing LSL-*Kras*^G12D/+^ mice (The Jackson Laboratory) with *Trp53*^*fl/fl*^ mice (The Jackson Laboratory). In all in vivo experiments, mice were blindly randomized before allocation to treatment groups as noted in the figure legends and below. This was achieved by pooling and then randomly separating mice into new cages. This allocation occurred at the 2-week time point following TVI of tumor cells before randomization to treatment groups. In tumor bioluminescence tracking experiments, outliers, if present, were reallocated to ensure equal means of tumor bioluminescence. Tumor quantification was performed on hematoxylin and eosin (H&E)-stained slides of formalin-fixed paraffin-embedded 4-µm lung tissue sections. Slides were scanned using an Olympus digital scanner and analyzed using the Panoramic viewer and QuPath software. As this study uses an orthotopic lung metastasis model, tumor and size could not be measured by external caliper. Tumor burden was monitored by weekly BLI when possible. Mice were monitored daily for signs of morbidity. Humane endpoints, as defined by the IACUC of the Icahn School of Medicine at Mount Sinai, included body weight loss exceeding 20% of pretreatment weight, labored breathing or respiratory distress, hunched posture, reduced mobility, failure to groom or body condition score of 2 or below^75^. Mice meeting any of these criteria were killed by CO_2_ inhalation.

### TRT

Mice were deeply anesthetized using intraperitoneal injection of a mixture of ketamine (100 mg kg^−1^) and xylazine (10 mg kg^−1^). Anesthetized mice were placed supine on an aluminum shelf plate under a cone-shaped irradiation field in a RS2000 small animal irradiator (Rad Source Technologies) that delivered 160 kV photons at 25 mA, with a dose rate of 2.1 Gy min^−1^.

Mice were treated with a half-beam block technique eliminating divergence into the neck superiorly. Radiation was delivered to the thorax through an aperture formed between two 2-mm lead sheets suspended above the mice, which shielded the head and neck superior to the apex of the thorax and tissues inferior to the xiphoid process. Following irradiation, mice were kept warm under a heat lamp and monitored until ambulatory.

### Lentiviral vector construction, production and transduction of cell lines

Transfer lentiviral plasmid was generated by cloning target sequences using standard molecular biology techniques into a self-inactivating lentiviral plasmid (pLM backbone)^73^. Human *CD19* cDNA was cloned in pLM-lentiviral vector backbone under the control of the PGK promoter. The hCD19–eGFP fusion was generated by fusing the eGFP sequence at the C terminus of CD19 using a GS linker and cloned into the pLM-lentiviral vector backbone as described previously^81^. VSV-G pseudotyped lentiviral supernatants derived from transfected 293T cells (ATCC, CRL-3216) were used to transduce KP cells (see above) to generate KP19 cells expressing human CD19. Transduction was performed in six-well plates containing 4 μg ml^−1^ polybrene.

### Genetic modification of T cells

Plasmids encoding the SFGγ retroviral vector^77^ were prepared using standard molecular biology techniques as described previously^78^. VSV-G pseudotyped retroviral supernatants derived from transduced gpg29 fibroblasts (H29) were used to construct stable retroviral-producing cell lines as described previously^63,78^.

SFG-h1928ζ comprises an scFv specific for human CD19 derived from the heavy chain (VH) and light chain (VL) variable regions from hybridoma cell line SJ25C1, which was cloned into an all-murine CD28/CD3ζ-based CAR SFG construct. The SFG–m1928ζ construct comprises a scFv specific for the mouse CD19 derived from the VH and VL chain variable regions from hybridoma cell line 1D3 as previously described^79^. To generate EpCAM CAR, an EpCAM-specific scFv, G8.8, was synthesized in VH–VL format with an N-terminal mouse CD8a signal peptide and MYC tag and cloned into the mouse CD28/CD3ζ-based CAR SFG construct. This design was also applied to generate GD2 and CD105 CAR, whereby scFvs specific to these antigens were synthesized in VH–VL format on the basis of published sequences of monoclonal antibody 14G2a (ref. ^80^) and MJ7/18 (ref. ^76^), respectively, and cloned into an all-murine CD28/CD3ζ-based CAR SFG construct.

The scFv regions are preceded by a mouse CD8A leader peptide and followed by the Myc tag sequence (EQKLISEEDL), mouse CD28 transmembrane and intracellular domains and mouse CD3ζ intracellular domain^63^. The SFG–CAR-2A–DsRed and SFG–CAR-2A–mCherryNLS vectors were constructed by Gibson assembly of the CAR gene without the stop codon to DsRed or mCherryNLS with a P2A self-cleaving peptide sequence (GSGATNFSLLKQAGDVEENPGP)^81^.

Spleens from killed 6-week-old mice were crushed and strained, the red blood cells (RBCs) were lysed and T cells were enriched by negative selection using the Pan T Cell Isolation Kit II, mouse (Miltenyi Biotec). Cells were then expanded in vitro by culturing in RPMI 1640 (Gibco, 11875093) supplemented with FBS (10%), penicillin–streptomycin (100 U per ml), sodium pyruvate (1 mM), HEPES (10 mM), β-mercaptoethanol (Gibco, 21985-023), MEM nonessential amino acids (13; Gibco, 11140050), 50 IU of recombinant human IL-2 (Peprotech, 200-02) and anti-CD3/28 Dynabeads (Life Technologies) at a bead-to-cell ratio of 1:2. Then, 24 h after initiating T cell activation, T cells were transduced with retroviral supernatants by centrifugation on RetroNectin-coated plates (Takara), as described previously^82^. Transduction efficiencies were determined 4 days later by flow cytometry and CARs were injected into tumor-bearing mice or used for in vitro experiments. For T cell imaging studies, mouse T cells were also transduced with retroviral supernatants encoding SFG–GFP–CBR-Luciferase^20,83^.

### BLI and quantification

For bioluminescence tumor studies, mice were inoculated i.v. with 5 × 10^5^ CBR-Luciferase^+^ KP19 tumor cells on day 0 and imaged weekly until mice reached humane endpoints.

For tracking of CBR-Luciferase^+^ CAR T cells, mice inoculated with KP19 tumor cells 3 weeks beforehand were randomized to treatment groups before undergoing lymphodepletion and i.v. injection of CBR-Luciferase^+^ CAR T cells.

Cohorts were then imaged 3 days after i.v. CAR T cell injections and weekly thereafter until mice reached humane endpoints.

Mice were injected with 1.5 mg of Luciferin diluted in PBS per 20 g of body weight through retro-orbital injection and imaged immediately after. BLI was performed using the Xenogen in vivo imaging system (Xenogen) with Living Image software (Xenogen). Acquisition of imaging datasets was conducted to guarantee equal tumor burden of mice at the time of treatment. Mice were then randomized into different treatment cohorts before CAR T cell therapy.

### Cytotoxic lymphocyte assay

A total of 50,000 GFP–Luciferase-expressing clonal target^+^ KP cells or B16-F10 melanoma cells were trypsinized and added to 96-well clear-bottom plates containing serial dilutions of CAR T cells and cocultured for 18 h after a brief centrifugation at 300*g* for 1 min. Then, 0.15 mg Luciferin in PBS was added to cocultures immediately before acquisition of luminescence on plate reader. For irradiation of cell lines, culture plates at 70% confluency were irradiated on the top-shelf plate under a cone-shaped irradiation field in the Rad Source RS2000 irradiator.

### Adoptive transfer of CAR T cells

Mice were lymphodepleted using 200 mg kg^−1^ cyclophosphamide (Sigma) intraperitoneally 3–4 days before adoptive transfer of 2.5 × 10^6^–4 × 10^6^ CAR^+^ T cells i.v. as indicated.

### Flow cytometry and FACS

Mice were killed with CO_2_ and single-cell suspensions from perfused mouse lungs were obtained upon lung tissue digestion with collagenase IV (0.25 mg ml^−1^; Sigma) at 37 °C for 30 min in agitation (80 r.p.m.) followed by passing through a 70-μm cell strainer and ammonium chloride–potassium lysing buffer (Lonza) for 5 min at room temperature. For flow cytometry or FACS, cells were stained in FACS buffer (PBS supplemented with 2% BSA and 5 mM EDTA) with different combinations of the following monoclonal antibodies: CD45 (clone 30-F11, BioLegend, 103137; 1:800 dilution), CD45.1 (clone A20, BioLegend; 1:200 dilution), B220 (clone RA3-6B, BioLegend; 1:100 dilution), human CD19 (clone HIB19, BioLegend; 1:50 dilution), Ly6G (clone 1A8, BioLegend, 127621; 1:200 dilution), CD64 (clone X54-5/7.1, BioLegend; 1:200 dilution), MERTK (clone 2B10C42, BioLegend; 1:200 dilution), CD2 (clone RM2-5, BioLegend, 100113; 1:200 dilution), Siglec-F (clone E50-2440, BD Pharmingen, 740956; 1:200 dilution), MHC-I-A/I-E (clone M5/114.15.2, eBiosciences, 12-5321-82; 1:400 dilution), CD11b (clone M1/70, eBiosciences, 45-0112-82; 1:200 dilution), CD11c (clone N418, Invitrogen, 47-0114-82; 1:250 dilution), XCR1 (clone ZET, BioLegend; 1:250 dilution), CD3 (17A2, BioLegend; 1:200 dilution), CD8a (clone 53-6.7, BioLegend, 558106; 1:1,000 dilution), CD4 (clone GK1.5, eBiosciences, 17-0041-82; 1:1,000 dilution), CD44 (clone IM7, BioLegend; 1:200 dilution), PD1 (clone RMP1-30, BioLegend; 1:200 dilution) and TIGIT (clone Vstm3, BioLegend; 1:200 dilution). For flow cytometry, cells were analyzed in a BD LSR Fortessa analyzer or CYTEK Aurora. For FACS, cells were prepared, stained and purified using a BD FACSAria sorter or Beckman Coulter CytoFLEX SRT cell sorter. Flow cytometry data were acquired using FACS Diva software version 7 (BD) and the data obtained were analyzed using FlowJo.

### Imaging and quantification of CAR T cells in mouse tissue sections

Mice were killed with CO_2_ and the left mouse lung was dissected after perfusion of the mouse lungs with 10 ml of PBS through the right ventricle. Formalin-fixed paraffin-embedded tissue sections (4 μm) of the lungs were stained using immunohistochemical staining as previously described^84^. In brief, slides were baked at 50 °C overnight, deparaffinized in xylene and rehydrated in decreasing concentrations of ethanol (100%, 90%, 70%, 50% and distilled H_2_O). Sample slides were incubated in pH 9 buffers at 95 °C for 30 min for antigen retrieval, then in 3% hydrogen peroxide for 15 min and finally in serum-free protein block solution (Dako) for 30 min. Primary antibody staining was performed using a 1:200 dilution for 1 h at room temperature or at 4 °C overnight, followed by signal amplification using associated secondary antibody conjugated to horseradish peroxidase for 30 min. Chromogenic revelation was performed using AEC (Vector). Tissue sections were counterstained with hematoxylin, mounted with a glycerol-based mounting medium and finally scanned to obtain digital images (Aperio AT2, Leica). DsRed was detected using anti-RFP (NBP1-69962 Novus Biologicals).

The tumor region of interest (ROI) was distinguished from lung parenchyma on corresponding paraffin sections stained with H&E. Nodules >40,000 µm^2^ were selected for quantification of intratumoral CAR T cell density. Cell segmentation was performed using StarDist extension (https://github.com/ksugar/stardist-sparse). Sample training images were used to train the system for accurate positive cell detection. Quantification of positively stained cells was successively performed on the ROIs using the trained algorithm and annotations were exported for downstream analysis.

### Nongenotoxic BM transplantation

Nongenotoxic BM transplantation was performed as previously described^37^. In brief, biotinylated anti-CD117 (BioLegend, clone 2B8, lot B376226) was combined with streptavidin–saporin conjugate (IT-27, lot 201-151, Advanced Targeting Systems) in a 1:1 molar ratio and then diluted in PBS to desired concentration. CD117–saporin conjugates were administered at 1 mg kg^−1^ (~10 µg of streptavidin–saporin) per recipient to selectively deplete host hematopoietic stem and progenitor cells. In vivo administration of immunotoxin was performed by i.v. injections (300 μl).

Donor BM cells were obtained by canulating and flushing femurs and tibias isolated from *Zbtb46*^*DTR/DTR*^ mice and resuspended in PBS after RBC lysis. BM cell numbers were determined on Nexcelom slides on a Cellometer Auto 2000. Next, 10 × 10^6^ BM cells were transplanted through retro-orbital i.v. infusion 5 days after CD117–saporin conjugate injection. Donor BM cells were allowed to repopulate the hosts for 34 days before the mice were injected with 7 × 10^5^ target^+^ KP adenocarcinoma cells through TVI.

### DT injections

Unnicked DT from *Corynebacterium* *diphtheriae* was purchased from List Biological Laboratories. *Zbtb46*^*DTR/DTR*^ BM chimeras were injected intraperitoneally with a first dose of 640 ng DT per mouse, followed by injections of 400 ng per mouse every 2–3 days (Monday, Wednesday and Friday).

### Live-cell imaging of CAR T cells and DCs in ex vivo cocultures

C57BL/6 mice were injected with 1 × 10^6^ target^+^ KP tumor cells through the tail vein and whole lungs were perfused and digested to generate single-cell suspensions as above. XCR1^+^CD11b^*−*^ DC1 and CD11b^+^XCR1^*−*^ DC2 populations were sorted from live CD45^+^Ly6G^*−*^B220^*−*^MERTK^*−*^CD11c^hi^MHCII^hi^ cells. Syngeneic T cells transduced with CAR-2A-mCherryNLS constructs as above were cocultured in 96-well flat-bottom plates at a 10:1 ratio with DC1 or DC2 populations sorted the same day from target^+^ KP tumor-bearing lungs or with DC medium alone. DC medium consisted of RPMI 1640 (Gibco, 11875093) supplemented with FBS (10%), penicillin–streptomycin (100 U per ml), β-mercaptoethanol (Gibco, 21985-023) and 100 ng ml^−1^ final concentration of recombinant human FLT3L (carrier-free formulation, BioLegend). Plates were briefly centrifuged and then imaged at 6-h intervals for mCherryNLS fluorescence using the BioTek Cytation 7 cell imaging multimode reader (Agilent) integrated with the BioTek BioSpa 8 automated incubator (Agilent). The accompanying Gen5 software (Agilent) was used to count the number of mCherryNLS-positive CAR T cells over four nonoverlapping ×10 widefield images captured per 96-well chamber for each time point.

### RNA sequencing

For bulk sequencing, CD4 and CD8 h1928ζ-2A-DsRed CAR T cells were sorted using the CytoFLEX SRT (Beckman Coulter) into 200 μl of TRIzol LS (Invitrogen, 10296010) in DNA LoBind tubes (Eppendorf, 022431021) precoated with FBS with 1× β-mercaptoethanol (Gibco, 21985-023). Cells were flash-frozen and submitted to Azenta Life Sciences for RNA extraction, library preparation and bulk sequencing. Quality control, trimming and alignment to produce a gene expression matrix from FASTQ files were conducted through the Mount Sinai Human Immune Monitoring Core (HIMC). The sequencing data were processed using the nf-core RNA-seq pipeline (https://nf-co.re/rnaseq). In brief, we performed quality control and trimming of the raw FASTQ files using FastQC and Trim Galore software, respectively. The processed FASTQ files were aligned to the *Mus* *musculus* genome using STAR. Salmon was used to generate a gene-by-sample count matrix for further analysis. Variance stabilization transformation was performed to produce read counts and principal component analysis was successively performed. The count data of the transcripts were normalized using the Bioconductor package DESeq2. Analysis of differentially expressed genes (DEGs) was also performed using the DESeq2 package with a *P*-value threshold of <0.05. Genes of interest were aggregated and their expression was standardized with *z* scores across the samples to produce the heat map. We performed over-representation analysis (ORA) using the web-based gene set analysis toolkit (WEBGestalt) version 2019 (GSE263352).

For 10x single-cell sequencing, the CD11b^+^CD11c^+^ double-positive fraction was sorted from the CD45^+^CD3^*−*^mCD19^*−*^Ly6G^*−*^ fraction of the single-cell suspension from tumor-bearing lungs 10 days after radiotherapy was delivered to half the cohort. Mice were irradiated 3 weeks after TVI of target^+^ KP cells. Both cohorts underwent lymphodepletion 7 days before sorting. Live cells were submitted to the Mount Sinai HIMC for single-cell sequencing on the 10x platform. Libraries processed with Cell Ranger version 6.1.2 and aligned to reference refdata-gex-mm10-2020-A (GSE316247).

### BMDC coculture

To generate mouse BMDCs, BM precursors were cultured in IMDM medium supplemented with 10% fetal calf serum, 20 mM glutamine, 100 U per ml penicillin–streptomycin and 50 μM β-mercaptoethanol and supplemented with granulocyte-macrophage colony-stimulating factor at a concentration of 50 ng ml^−1^, derived from supernatant obtained from transfected J558 cells, for a period of 9 days, as per established protocols. Immature DCs were isolated by gently retrieving semiadherent cells from the culture dishes and added to vials containing nonenzymatically digested tumors cells (using Cellstripper, Corning) at a ratio of 1:10.

For confocal imaging of antigen-dressed BMDCs, BMDCs were cultured on glass coverslips and fixed by incubating for 15 min on ice in 3.7% w/v paraformaldehyde. The cells were then quenched for 10 min with 50 mM NH_4_Cl in PBS. Subsequently, the cells were incubated for 1 h with specific primary antibodies in PBS, washed twice and incubated for 1 h with fluorescently labeled secondary antibodies. Finally, the cells were mounted on slides using Prolong Gold antifade reagent with DAPI. Confocal microscopy was performed using a Zeiss LSM 780 system (Carl Zeiss) with ×63 objectives. Image processing was carried out with Zeiss LSM Image Browser (Carl Zeiss) and ImageJ software.

### Statistics analysis

No statistical methods were used to determine sample sizes, although preliminary experiments were leveraged to estimate appropriate sample sizes, with an effort to achieve a minimum of *n* = 4 mice per experimental group per experiment, which proved sufficient to ascertain reproducible and biologically relevant results. The sample sizes reported in the present study are comparable to those used in previous and similar publications^16,17^. All experiments were performed with distinct biological replicates for in vivo studies and further validated with technical replicates for in vitro experiments, including coculture experiments, as indicated in the text and in the figure legends. For in vivo studies, in particular, variation amongst mice that were inoculated with tumor cells was taken into consideration.

Data collection and analysis were not performed blind to the conditions of the experiments. For biological experiments, mice that met endpoint criteria before initiating CAR T cell therapy were excluded from survival analyses. Mice that met endpoint because of tumor growth outside the lungs (that is facial or tail nodules) were excluded from survival analyses. All in vivo irradiation experiments were performed in female mice to avoid aggression-related injury in mixed-sex housing of randomized cohorts. Both the KP lung adenocarcinoma and B16-F10 melanoma models were established and characterized in female mice accordingly. Sex was, therefore, not considered as a variable in the study design.

A two-sided Student’s *t*-test was used for determination of statistical significance unless indicated otherwise in figure legends. Shapiro–Wilk tests were used to confirm normality with α = 0.05 for data where the Student’s *t*-test was used for pairwise comparisons. Survival curves were generated using the Kaplan–Meier method and differences among groups were assessed with the log-rank (Mantel–Cox) test to evaluate the overall significance of survival distributions (*P* < 0.05 indicating a global difference across all groups). Bars show median values unless otherwise noted.

### Reporting summary

Further information on research design is available in the Nature Portfolio Reporting Summary linked to this article.

## Supplementary information

Reporting Summary

Peer Review File

Supplementary Table Supplementary Table 1. Bulk RNA sequencing of CAR T cells sorted from irradiated versus unirradiated tumors. DESeq2 differential gene expression analysis of CD8^+^ and CD4^+^ CAR T cells sorted from irradiated versus unirradiated KP19 tumor-bearing lungs (related to Fig. 2). Gene-level information includes Ensembl ID, gene biotype, description and genomic coordinates (chromosome, start, end, strand and transcription start site) for 13,277 genes. Three normalized expression matrices are provided across separate tabs: counts per million, variance-stabilizing transformation and regularized log transformation. Each tab contains per-sample expression values for CD8^+^ CAR T cells (*n* = 5 per condition) and pooled CD4^+^ CAR T cells, average expression per group and DESeq2 results (log_2_ fold change, *P* value and false discovery rate) for CD8^+^ irradiated versus unirradiated and CD4^+^ irradiated versus unirradiated comparisons.

## Source data

Source Data Fig. 1 Statistical source data.

Source Data Fig. 2 Statistical source data.

Source Data Fig. 3 Statistical source data.

Source Data Fig. 4 Statistical source data.

Source Data Fig. 5 Statistical source data.

Source Data Fig. 6 Statistical source data.

Source Data Extended Data Fig. 1 Statistical source data.

Source Data Extended Data Fig. 2 Statistical source data.

Source Data Extended Data Fig. 3 Statistical source data.

Source Data Extended Data Fig. 4 Statistical source data.

Source Data Extended Data Fig. 5 Statistical source data.

Source Data Extended Data Fig. 6 Statistical source data.

## Data Availability

Bulk RNA-seq data and single-cell RNA-seq data were deposited to the Gene Expression Omnibus (GEO) under accession codes GSE263352 and GSE316247, respectively. All other data supporting the findings of this study are available within the article and its Supplementary Information. All other data supporting the findings of this study are available from the corresponding author on reasonable request. Materials that are subject to existing intellectual property obligations will be made available upon execution of a material transfer agreement. Source data are provided with this paper.

## References

[1] Park, J. H. et al. Long-term follow-up of CD19 CAR therapy in acute lymphoblastic leukemia. *N. Engl. J. Med.* **378**, 449–459 (2018). 29385376 10.1056/NEJMoa1709919 PMC6637939

[2] Maude, S. L. et al. Tisagenlecleucel in children and young adults with B-cell lymphoblastic leukemia. *N. Engl. J. Med.* **378**, 439–448 (2018). 29385370 10.1056/NEJMoa1709866 PMC5996391

[3] Hubbeling, H. et al. Metabolic tumor volume response after bridging therapy determines chimeric antigen receptor T-cell outcomes in large B-cell lymphoma. *Clin. Cancer Res.* **30**, 5083–5093 (2024). 39259292 10.1158/1078-0432.CCR-24-0830 PMC12001374

[4] Albelda, S. M. CAR T cell therapy for patients with solid tumours: key lessons to learn and unlearn. *Nat. Rev. Clin. Oncol.* **21**, 47–66 (2024). 37904019 10.1038/s41571-023-00832-4

[5] Sadelain, M., Riviere, I. & Riddell, S. Therapeutic T cell engineering. *Nature* **545**, 423–431 (2017). 28541315 10.1038/nature22395 PMC5632949

[6] Morgan, R. A. et al. Case report of a serious adverse event following the administration of T cells transduced with a chimeric antigen receptor recognizing ERBB2. *Mol. Ther.* **18**, 843–851 (2010). 20179677 10.1038/mt.2010.24 PMC2862534

[7] Haas, A. R. et al. Phase I study of lentiviral-transduced chimeric antigen receptor-modified T cells recognizing mesothelin in advanced solid cancers. *Mol. Ther.* **27**, 1919–1929 (2019). 31420241 10.1016/j.ymthe.2019.07.015 PMC6838875

[8] Jürgens, B. & Clarke, N. S. Evolution of CAR T-cell immunotherapy in terms of patenting activity. *Nat. Biotechnol.* **37**, 370–375 (2019). 30940940 10.1038/s41587-019-0083-5

[9] Anderson, K. G., Stromnes, I. M. & Greenberg, P. D. Obstacles posed by the tumor microenvironment to T cell activity: a case for synergistic therapies. *Cancer Cell* **31**, 311–325 (2017). 28292435 10.1016/j.ccell.2017.02.008 PMC5423788

[10] Lapteva, N. et al. T-cell receptor stimulation enhances the expansion and function of CD19 chimeric antigen receptor-expressing T cells. *Clin. Cancer Res.* **25**, 7340–7350 (2019). 31558475 10.1158/1078-0432.CCR-18-3199 PMC7062259

[11] Evgin, L. et al. Oncolytic virus-mediated expansion of dual-specific CAR T cells improves efficacy against solid tumors in mice. *Sci. Transl. Med.* **14**, eabn2231 (2022). 35417192 10.1126/scitranslmed.abn2231 PMC9297825

[12] Reinhard, K. et al. An RNA vaccine drives expansion and efficacy of claudin-CAR-T cells against solid tumors. *Science* **367**, 446–453 (2020). 31896660 10.1126/science.aay5967

[13] Ma, L. et al. Enhanced CAR-T cell activity against solid tumors by vaccine boosting through the chimeric receptor. *Science* **365**, 162–168 (2019). 31296767 10.1126/science.aav8692 PMC6800571

[14] Mackensen, A. et al. CLDN6-specific CAR-T cells plus amplifying RNA vaccine in relapsed or refractory solid tumors: the phase 1 BNT211-01 trial. *Nat. Med.* **29**, 2844–2853 (2023). 37872225 10.1038/s41591-023-02612-0 PMC10667102

[15] Bejcek, B. E. et al. Development and characterization of three recombinant single chain antibody fragments (scFvs) directed against the CD19 antigen. *Cancer Res.* **55**, 2346–2351 (1995). 7538901

[16] Maier, B. et al. A conserved dendritic-cell regulatory program limits antitumour immunity. *Nature* **580**, 257–262 (2020). 32269339 10.1038/s41586-020-2134-y PMC7787191

[17] Casanova-Acebes, M. et al. Tissue-resident macrophages provide a pro-tumorigenic niche to early NSCLC cells. *Nature* **595**, 578–584 (2021). 34135508 10.1038/s41586-021-03651-8 PMC8923521

[18] Jackson, I. L. et al. A preclinical rodent model of radiation-induced lung injury for medical countermeasure screening in accordance with the FDA animal rule. *Health Phys.* **103**, 463–473 (2012). 22929472 10.1097/HP.0b013e31826386ef PMC3604892

[19] Hartsell, W. F. et al. Randomized trial of short- versus long-course radiotherapy for palliation of painful bone metastases. *J. Natl Cancer Inst.* **97**, 798–804 (2005). 15928300 10.1093/jnci/dji139

[20] Amor, C. et al. Senolytic CAR T cells reverse senescence-associated pathologies. *Nature* **583**, 127–132 (2020). 32555459 10.1038/s41586-020-2403-9 PMC7583560

[21] Kostopoulos, N. et al. Local radiation enhances systemic CAR T-cell efficacy by augmenting antigen crosspresentation and T-cell infiltration. *Blood Adv.* **8**, 6308–6320 (2024). 39213422 10.1182/bloodadvances.2024012599 PMC11700247

[22] Sugita, M. et al. Radiation therapy improves CAR T cell activity in acute lymphoblastic leukemia. *Cell Death Dis.* **14**, 305 (2023). 37142568 10.1038/s41419-023-05829-6 PMC10160073

[23] Heylmann, D., Rödel, F., Kindler, T. & Kaina, B. Radiation sensitivity of human and murine peripheral blood lymphocytes, stem and progenitor cells. *Biochim. Biophys. Acta* **1846**, 121–129 (2014). 24797212 10.1016/j.bbcan.2014.04.009

[24] Gerszten, P. C., Mendel, E. & Yamada, Y. Radiotherapy and radiosurgery for metastatic spine disease. *Spine* **34**, S78–S92 (2009). 19829280 10.1097/BRS.0b013e3181b8b6f5

[25] Tassi, E. et al. Early effector T lymphocytes coexpress multiple inhibitory receptors in primary non-small cell lung cancer. *Cancer Res.* **77**, 851–861 (2017). 27979840 10.1158/0008-5472.CAN-16-1387

[26] Sorin, M. et al. Single-cell spatial landscapes of the lung tumour immune microenvironment. *Nature* **614**, 548–554 (2023). 36725934 10.1038/s41586-022-05672-3 PMC9931585

[27] Reinfeld, B. I. et al. Cell-programmed nutrient partitioning in the tumour microenvironment. *Nature* **593**, 282–288 (2021). 33828302 10.1038/s41586-021-03442-1 PMC8122068

[28] Park, M. D. et al. TREM2 macrophages drive NK cell paucity and dysfunction in lung cancer. *Nat. Immunol.* **24**, 792–801 (2023). 37081148 10.1038/s41590-023-01475-4 PMC11088947

[29] Chow, A. et al. Bone marrow CD169^+^ macrophages promote the retention of hematopoietic stem and progenitor cells in the mesenchymal stem cell niche. *J. Exp. Med.* **208**, 261–271 (2011). 21282381 10.1084/jem.20101688 PMC3039855

[30] Hashimoto, D. et al. Tissue-resident macrophages self-maintain locally throughout adult life with minimal contribution from circulating monocytes. *Immunity* **38**, 792–804 (2013). 23601688 10.1016/j.immuni.2013.04.004 PMC3853406

[31] Cabeza-Cabrerizo, M., Cardoso, A., Minutti, C. M., Pereira da Costa, M. & Reis e Sousa, C. Dendritic cells revisited. *Annu. Rev. Immunol.* **39**, 131–166 (2021). 33481643 10.1146/annurev-immunol-061020-053707

[32] Böttcher, J. P. et al. NK cells stimulate recruitment of cDC1 into the tumor microenvironment promoting cancer immune control. *Cell* **172**, 1022–1037 (2018). 29429633 10.1016/j.cell.2018.01.004 PMC5847168

[33] Anderson, D. A., Dutertre, C.-A., Ginhoux, F. & Murphy, K. M. Genetic models of human and mouse dendritic cell development and function. *Nat. Rev. Immunol.* **21**, 101–115 (2021). 32908299 10.1038/s41577-020-00413-x PMC10955724

[34] Ginhoux, F., Guilliams, M. & Merad, M. Expanding dendritic cell nomenclature in the single-cell era. *Nat. Rev. Immunol.* **22**, 67–68 (2022). 35027741 10.1038/s41577-022-00675-7

[35] Satpathy, A. T. et al. *Zbtb46* expression distinguishes classical dendritic cells and their committed progenitors from other immune lineages. *J. Exp. Med.* **209**, 1135–1152 (2012). 22615127 10.1084/jem.20120030 PMC3371733

[36] Meredith, M. M. et al. Expression of the zinc finger transcription factor zDC (Zbtb46, Btbd4) defines the classical dendritic cell lineage. *J. Exp. Med.* **209**, 1153–1165 (2012). 22615130 10.1084/jem.20112675 PMC3371731

[37] Czechowicz, A. et al. Selective hematopoietic stem cell ablation using CD117-antibody–drug-conjugates enables safe and effective transplantation with immunity preservation. *Nat. Commun.* **10**, 617 (2019). 30728354 10.1038/s41467-018-08201-x PMC6365495

[38] Davis, D. M. Intercellular transfer of cell-surface proteins is common and can affect many stages of an immune response. *Nat. Rev. Immunol.* **7**, 238–243 (2007). 17290299 10.1038/nri2020

[39] Hildner, K. et al. Batf3 deficiency reveals a critical role for CD8α^+^ dendritic cells in cytotoxic T cell immunity. *Science* **322**, 1097–1100 (2008). 19008445 10.1126/science.1164206 PMC2756611

[40] Spranger, S., Dai, D., Horton, B. & Gajewski, T. F. Tumor-residing Batf3 dendritic cells are required for effector T cell trafficking and adoptive T cell therapy. *Cancer Cell* **31**, 711–723 (2017). 28486109 10.1016/j.ccell.2017.04.003 PMC5650691

[41] Salmon, H. et al. Expansion and activation of CD103^+^ dendritic cell progenitors at the tumor site enhances tumor responses to therapeutic PD-L1 and BRAF inhibition. *Immunity* **44**, 924–938 (2016). 27096321 10.1016/j.immuni.2016.03.012 PMC4980762

[42] Majzner, R. G. et al. GD2-CAR T cell therapy for H3K27M-mutated diffuse midline gliomas. *Nature* **603**, 934–941 (2022). 35130560 10.1038/s41586-022-04489-4 PMC8967714

[43] Mount, C. W. et al. Potent antitumor efficacy of anti-GD2 CAR T cells in H3-K27M^+^ diffuse midline gliomas. *Nat. Med.* **24**, 572–579 (2018). 29662203 10.1038/s41591-018-0006-x PMC6214371

[44] Went, P. T. H. et al. Frequent EpCam protein expression in human carcinomas. *Hum. Pathol.* **35**, 122–128 (2004). 14745734 10.1016/j.humpath.2003.08.026

[45] Moldenhauer, G., Momburg, F., Möller, P., Schwartz, R. & Hämmerling, G. Epithelium-specific surface glycoprotein of Mr 34,000 is a widely distributed human carcinoma marker. *Br. J. Cancer* **56**, 714–721 (1987). 2449234 10.1038/bjc.1987.276 PMC2002400

[46] Qin, D. et al. Potential lung attack and lethality generated by EpCAM-specific CAR-T cells in immunocompetent mouse models. *OncoImmunology* **9**, 1806009 (2020). 32923168 10.1080/2162402X.2020.1806009 PMC7458607

[47] Srivastava, S. et al. Immunogenic chemotherapy enhances recruitment of CAR-T cells to lung tumors and improves antitumor efficacy when combined with checkpoint blockade. *Cancer Cell* **39**, 193–208 (2021). 33357452 10.1016/j.ccell.2020.11.005 PMC7878409

[48] Mani, K. et al. Causes of death among people living with metastatic cancer. *Nat. Commun.* **15**, 1519 (2024). 38374318 10.1038/s41467-024-45307-x PMC10876661

[49] Siegel, R. L., Kratzer, T. B., Giaquinto, A. N., Sung, H. & Jemal, A. Cancer statistics, 2025. *CA Cancer J. Clin.* **75**, 10–45 (2025). 39817679 10.3322/caac.21871 PMC11745215

[50] Guisier, F., Cousse, S., Jeanvoine, M., Thiberville, L. & Salaun, M. A rationale for surgical debulking to improve anti-PD1 therapy outcome in non small cell lung cancer. *Sci. Rep.* **9**, 16902 (2019). 31729430 10.1038/s41598-019-52913-z PMC6858444

[51] Joseph, R. W. et al. Baseline tumor size is an independent prognostic factor for overall survival in patients with melanoma treated with pembrolizumab. *Clin. Cancer Res.* **24**, 4960–4967 (2018). 29685882 10.1158/1078-0432.CCR-17-2386 PMC6916264

[52] Hanson, H. L. et al. Eradication of established tumors by CD8^+^ T cell adoptive immunotherapy. *Immunity* **13**, 265–276 (2000). 10981969 10.1016/s1074-7613(00)00026-1

[53] Sánchez-Paulete, A. R. et al. Targeting macrophages with CAR T cells delays solid tumor progression and enhances anti-tumor immunity. *Cancer Immunol. Res.* **10**, 1354–1369 (2022). 36095236 10.1158/2326-6066.CIR-21-1075 PMC10704925

[54] D’Angelo, C. R. et al. A prospective clinical trial of boom-boom radiation as bridging therapy for patients with large B-cell lymphoma undergoing treatment with lisocabtagene maraleucel (liso-cel) therapy. *Blood* **144**, 3126–3126 (2024).

[55] Ban, Y. et al. Radiation-activated secretory proteins of Scgb1a1^+^ club cells increase the efficacy of immune checkpoint blockade in lung cancer. *Nat. Cancer* **2**, 919–931 (2021). 34917944 10.1038/s43018-021-00245-1 PMC8670735

[56] Chang, J. Y. et al. Stereotactic ablative radiotherapy with or without immunotherapy for early-stage or isolated lung parenchymal recurrent node-negative non-small-cell lung cancer: an open-label, randomised, phase 2 trial. *Lancet* **402**, 871–881 (2023). 37478883 10.1016/S0140-6736(23)01384-3 PMC10529504

[57] Bassetti, M. F. et al. Combining dual checkpoint immunotherapy with ablative radiation to all sites of oligometastatic non-small cell lung cancer: toxicity and efficacy results of a phase 1b trial. *Int. J. Radiat. Oncol. Biol. Phys.* **118**, 1481–1489 (2024). 38072321 10.1016/j.ijrobp.2023.11.040 PMC10947887

[58] Brown, C. E. et al. Regression of glioblastoma after chimeric antigen receptor T-cell therapy. *N. Engl. J. Med.* **375**, 2561–2569 (2016). 28029927 10.1056/NEJMoa1610497 PMC5390684

[59] Mo, G. et al. Long-term remissions following CD20-directed chimeric antigen receptor–adoptive T-cell therapy. *Blood Cancer Discov.* **5**, 258–266 (2024). 38747505 10.1158/2643-3230.BCD-23-0263 PMC11215399

[60] Smith, E. L. et al. BCMA-targeted CAR T-cell therapy plus radiotherapy for the treatment of refractory myeloma reveals potential synergy. *Cancer Immunol. Res.* **7**, 1047–1053 (2019). 31113804 10.1158/2326-6066.CIR-18-0551 PMC6606365

[61] Alizadeh, D. et al. IFNγ is critical for CAR T cell-mediated myeloid activation and induction of endogenous immunity. *Cancer Discov.* **11**, 2248–2265 (2021). 33837065 10.1158/2159-8290.CD-20-1661 PMC8561746

[62] Conde, E. et al. Epitope spreading driven by the joint action of CART cells and pharmacological STING stimulation counteracts tumor escape via antigen-loss variants. *J. Immunother. Cancer* **9**, e003351 (2021). 34810235 10.1136/jitc-2021-003351 PMC8609946

[63] Kuhn, N. F. et al. CD40 ligand-modified chimeric antigen receptor T cells enhance antitumor function by eliciting an endogenous antitumor response. *Cancer Cell* **35**, 473–488 (2019). 30889381 10.1016/j.ccell.2019.02.006 PMC6428219

[64] Goff, S. L. et al. Randomized, prospective evaluation comparing intensity of lymphodepletion before adoptive transfer of tumor-infiltrating lymphocytes for patients with metastatic melanoma. *J. Clin. Oncol.* **34**, 2389–2397 (2016). 27217459 10.1200/JCO.2016.66.7220 PMC4981979

[65] Jung, S. et al. In vivo depletion of CD11c^+^ dendritic cells abrogates priming of CD8^+^ T cells by exogenous cell-associated antigens. *Immunity* **17**, 211–220 (2002). 12196292 10.1016/s1074-7613(02)00365-5 PMC3689299

[66] Molthoff, C. F. M. et al. Monitoring response to radiotherapy in human squamous cell cancer bearing nude mice: comparison of 2′-deoxy-2′-[^18^F]fluoro-d-glucose (FDG) and 3′-[^18^F]fluoro-3′-deoxythymidine (FLT). *Mol. Imaging Biol.* **9**, 340–347 (2007). 17643202 10.1007/s11307-007-0104-5 PMC2040178

[67] Kubota, K. et al. Tracer feasibility for monitoring tumor radiotherapy: a quadruple tracer study with fluorine-18-fluorodeoxyglucose or fluorine-18-fluorodeoxyuridine, L-[methyl-^14^C]methionine, [6-^3^H]thymidine, and gallium-67. *J. Nucl. Med.* **32**, 2118–2123 (1991). 1834814

[68] Maruyama, I. et al. Hyperacute changes in glucose metabolism of brain tumors after stereotactic radiosurgery: a PET study. *J. Nucl. Med.* **40**, 1085–1090 (1999). 10405124

[69] Rozental, J. M. et al. Early changes in tumor metabolism after treatment: the effects of stereotactic radiotherapy. *Int. J. Radiat. Oncol. Biol. Phys.* **20**, 1053–1060 (1991). 2022505 10.1016/0360-3016(91)90204-h

[70] Martino, M. D. et al. Radiation therapy promotes unsaturated fatty acids to maintain survival of glioblastoma. *Cancer Lett.* **570**, 216329 (2023). 37499741 10.1016/j.canlet.2023.216329

[71] Ni, H. et al. FLASH radiation reprograms lipid metabolism and macrophage immunity and sensitizes medulloblastoma to CAR-T cell therapy. *Nat. Cancer* **6**, 460–473 (2025). 39910249 10.1038/s43018-025-00905-6 PMC12244404

[72] Liu, X. & Peng, G. Mitochondria orchestrate T cell fate and function. *Nat. Immunol.* **22**, 276–278 (2021). 33495653 10.1038/s41590-020-00861-6 PMC12416820

[73] Papapetrou, E. P. et al. Genomic safe harbors permit high β-globin transgene expression in thalassemia induced pluripotent stem cells. *Nat. Biotechnol.* **29**, 73–78 (2011). 21151124 10.1038/nbt.1717 PMC3356916

[74] DuPage, M., Dooley, A. L. & Jacks, T. Conditional mouse lung cancer models using adenoviral or lentiviral delivery of Cre recombinase. *Nat. Protoc.* **4**, 1064–1072 (2009). 19561589 10.1038/nprot.2009.95 PMC2757265

[75] Ullman-Culleré, M. H. & Foltz, C. J. Body condition scoring: a rapid and accurate method for assessing health status in mice. *Lab. Anim. Sci.* **49**, 319–323 (1999). 10403450

[76] Lontos, K. et al. Fully murine CD105-targeted CAR-T cells provide an immunocompetent model for CAR-T cell biology. *OncoImmunology* **11**, 2131229 (2022). 36275862 10.1080/2162402X.2022.2131229 PMC9586682

[77] Rivière, I., Brose, K. & Mulligan, R. C. Effects of retroviral vector design on expression of human adenosine deaminase in murine bone marrow transplant recipients engrafted with genetically modified cells. *Proc. Natl Acad. Sci. USA* **92**, 6733–6737 (1995). 7624312 10.1073/pnas.92.15.6733 PMC41403

[78] Brentjens, R. J. et al. Eradication of systemic B-cell tumors by genetically targeted human T lymphocytes co-stimulated by CD80 and interleukin-15. *Nat. Med.* **9**, 279–286 (2003). 12579196 10.1038/nm827

[79] Davila, M. L., Kloss, C. C., Gunset, G. & Sadelain, M. CD19 CAR-targeted T cells induce long-term remission and B cell aplasia in an immunocompetent mouse model of B cell acute lymphoblastic leukemia. *PLoS ONE* **8**, e61338-14 (2013). 23585892 10.1371/journal.pone.0061338 PMC3621858

[80] Blaeschke, F. et al. Modular pooled discovery of synthetic knockin sequences to program durable cell therapies. *Cell* **186**, 4216–4234 (2023). 37714135 10.1016/j.cell.2023.08.013 PMC10508323

[81] Hamieh, M. et al. CAR T cell trogocytosis and cooperative killing regulate tumour antigen escape. *Nature* **568**, 112–116 (2019). 30918399 10.1038/s41586-019-1054-1 PMC6707377

[82] Lee, J., Sadelain, M. & Brentjens, R. Retroviral transduction of murine primary T lymphocytes. *Methods Mol. Biol.* **506**, 83–96 (2009). 19110621 10.1007/978-1-59745-409-4_7 PMC5003426

[83] Dobrenkov, K. et al. Monitoring the efficacy of adoptively transferred prostate cancer-targeted human T lymphocytes with PET and bioluminescence imaging. *J. Nucl. Med.* **49**, 1162–1170 (2008). 18552144 10.2967/jnumed.107.047324 PMC2756034

[84] Remark, R. et al. In-depth tissue profiling using multiplexed immunohistochemical consecutive staining on single slide. *Sci. Immunol.* **1**, aaf6925 (2016). 28783673 10.1126/sciimmunol.aaf6925 PMC10152404

