# Supplementary material for: Tumor irradiation promotes antigen dressing of dendritic cells to enhance CAR T cell persistence and efficacy in lung metastases
Source: Nat Cancer. 2026 May 22;7(7):1104–20. doi: 10.1038/s43018-026-01167-6 (PMC13400301; doi:10.1038/s43018-026-01167-6)
Supplement: Supplementary file 1 — Reporting Summary [file 43018_2026_1167_MOESM1_ESM.pdf]

nature portfolio

Corresponding author(s): Jalal Ahmed

Last updated by author(s): Mar 25, 2026

Reporting Summary

Nature Portfolio wishes to improve the reproducibility of the work that we publish. This form provides structure for consistency and transparency in reporting. For further information on Nature Portfolio policies, see our Editorial Policies and the Editorial Policy Checklist.

Statistics

For all statistical analyses, confirm that the following items are present in the figure legend, table legend, main text, or Methods section.

n/a	Confirmed	
☐	☒	The exact sample size (*n*) for each experimental group/condition, given as a discrete number and unit of measurement
☐	☒	A statement on whether measurements were taken from distinct samples or whether the same sample was measured repeatedly
☐	☒	The statistical test(s) used AND whether they are one- or two-sided<br>*Only common tests should be described solely by name; describe more complex techniques in the Methods section.*
☐	☒	A description of all covariates tested
☐	☒	A description of any assumptions or corrections, such as tests of normality and adjustment for multiple comparisons
☐	☒	A full description of the statistical parameters including central tendency (e.g. means) or other basic estimates (e.g. regression coefficient) AND variation (e.g. standard deviation) or associated estimates of uncertainty (e.g. confidence intervals)
☐	☒	For null hypothesis testing, the test statistic (e.g. F, t, r) with confidence intervals, effect sizes, degrees of freedom and P value noted<br>*Give P values as exact values whenever suitable.*
☒	☐	For Bayesian analysis, information on the choice of priors and Markov chain Monte Carlo settings
☒	☐	For hierarchical and complex designs, identification of the appropriate level for tests and full reporting of outcomes
☒	☐	Estimates of effect sizes (e.g. Cohen's *d*, Pearson's *r*), indicating how they were calculated

Our web collection on statistics for biologists contains articles on many of the points above.

Software and code

Policy information about availability of computer code

Data collection	Bioluminescence imaging was done using the Xenogen IVIS Imaging System (Xenogen) with Living Image software (Xenogen). Immunohistochemistry slides were scanned using an Olympus digital scanner and analyzed using the Panoramic viewer and QuPath software. BD Fortessa II and Cytek Aurora 5L cytometers were used to collect flow cytometry data. Confocal microscopy was performed using a Zeiss LSM 780 system (Carl Zeiss) with 63x objectives. Single-cell sequencing was performed on the 10X platform.
Data analysis	Graphpad Prism V10 and Microsoft Excel was used to perform statistical tests. Cell segmentation was performed using StarDist extension in the QuPath software (Github repository: https://github.com/ksugar/ stardist-sparse). Flow cytometry data were acquired using FACS Diva software v.7 5 (BD) and the data obtained were analyzed using FlowJo 10.1 (LLC). For live imaging of cells ex vivo in co-culture, Gen5 software (Agilent) was used to count the cells. Image processing was carried out with Zeiss LSM Image Browser (Carl Zeiss) and ImageJ software. The bulk RNA sequencing data was processed using the nf-core RNA-seq pipeline (https://nf-co.re/rnaseq) and analyzed using R version 4.2.2. Briefly, we performed quality control and trimming of the raw fastq-files using FastQC and Trim Galore software, respectively. The processed fastq files were aligned to the mus musculus genome using STAR. Salmon was used to generate a gene-by sample count matrix for further analysis. Variance stabilization transformation was performed to produce read counts, and PCA was successively performed. The count data of the transcripts were normalized using the Bioconductor package DESeq2. Differential gene expression analysis was also performed using the DESeq2 package with a threshold of P value less than 0.05. Single-cell sequencing libraries were processed with Cell Ranger v6.1.2 and aligned to reference refdata-gex-mm10-2020-A.

For manuscripts utilizing custom algorithms or software that are central to the research but not yet described in published literature, software must be made available to editors and reviewers. We strongly encourage code deposition in a community repository (e.g. GitHub). See the Nature Portfolio guidelines for submitting code & software for further information.

Data

Policy information about availability of data

All manuscripts must include a data availability statement. This statement should provide the following information, where applicable:

- Accession codes, unique identifiers, or web links for publicly available datasets
- A description of any restrictions on data availability
- For clinical datasets or third party data, please ensure that the statement adheres to our policy

Source data for Fig. 1-6 and Extended Data Fig. 1-6 have been provided as Source Data files. Bulk RNA-seq data and single-cell RNA-seq data have been deposited in the Gene Expression Omnibus (GEO). GEO accession codes for Bulk RNA sequencing data are GSE263352 provided at [https://www.ncbi.nlm.nih.gov/geo/query/acc.cgi?acc=GSE263352]
and single cell sequencing data GSE316247 at [https://www.ncbi.nlm.nih.gov/geo/query/acc.cgi?acc=GSE316247]. All other data supporting the findings of this study are available within the article and its supplementary information. All other data supporting the findings of this study are available from the corresponding author on reasonable request. Materials that are subject to existing intellectual property obligations will be made available upon execution of a material transfer agreement.

Research involving human participants, their data, or biological material

Policy information about studies with human participants or human data. See also policy information about sex, gender (identity/presentation), and sexual orientation and race, ethnicity and racism.

Reporting on sex and gender	*Use the terms sex (biological attribute) and gender (shaped by social and cultural circumstances) carefully in order to avoid confusing both terms. Indicate if findings apply to only one sex or gender; describe whether sex and gender were considered in study design; whether sex and/or gender was determined based on self-reporting or assigned and methods used. Provide in the source data disaggregated sex and gender data, where this information has been collected, and if consent has been obtained for sharing of individual-level data; provide overall numbers in this Reporting Summary. Please state if this information has not been collected. Report sex- and gender-based analyses where performed, justify reasons for lack of sex- and gender-based analysis.*
Reporting on race, ethnicity, or other socially relevant groupings	*Please specify the socially constructed or socially relevant categorization variable(s) used in your manuscript and explain why they were used. Please note that such variables should not be used as proxies for other socially constructed/relevant variables (for example, race or ethnicity should not be used as a proxy for socioeconomic status). Provide clear definitions of the relevant terms used, how they were provided (by the participants/respondents, the researchers, or third parties), and the method(s) used to classify people into the different categories (e.g. self-report, census or administrative data, social media data, etc.) Please provide details about how you controlled for confounding variables in your analyses.*
Population characteristics	*Describe the covariate-relevant population characteristics of the human research participants (e.g. age, genotypic information, past and current diagnosis and treatment categories). If you filled out the behavioural & social sciences study design questions and have nothing to add here, write "See above."*
Recruitment	*Describe how participants were recruited. Outline any potential self-selection bias or other biases that may be present and how these are likely to impact results.*
Ethics oversight	*Identify the organization(s) that approved the study protocol.*

Note that full information on the approval of the study protocol must also be provided in the manuscript.

Field-specific reporting

Please select the one below that is the best fit for your research. If you are not sure, read the appropriate sections before making your selection.

☒ Life sciences ☐ Behavioural & social sciences ☐ Ecological, evolutionary & environmental sciences

For a reference copy of the document with all sections, see nature.com/documents/nr-reporting-summary-flat.pdf

Life sciences study design

All studies must disclose on these points even when the disclosure is negative.

Sample size	No statistical methods were used to predetermine sample size. Sample sizes were estimate based on preliminary experiments and was set to 5-10 mice per group to account for the variability in tumor engraftment using the tail-vein injection technique. We made the effort to achieve a minimum samples size which proved to be sufficient to reproducibly observe significance.
Data exclusions	None
Replication	All experimental results were reproduced prior to assembly of the manuscript. Data compiled from multiple experiments are included. In vitro and in vivo experiments were replicated and cross-validated with complimentary approaches (i.e. Bioluminescence tumor or T cell tracking

validated with IHC or flow cytometry in separate cohorts)

Randomization

In all in vivo experiments, mice were blindly randomized prior to allocation to treatment groups as noted in legends and methods. This was done by pooling and then randomly separating mice into new cages. In tumor tracking experiments, outliers if present were reallocated allocated at the two week time point following TVI of tumor cells prior to randomization to treatment groups.

Blinding

Mice were tracked individually using ear tags and cage card identification to ensure treatment groups were not accidentally mixed. Therefore, blinding was not possible.

Reporting for specific materials, systems and methods

We require information from authors about some types of materials, experimental systems and methods used in many studies. Here, indicate whether each material, system or method listed is relevant to your study. If you are not sure if a list item applies to your research, read the appropriate section before selecting a response.

Materials & experimental systems

n/a	Involved in the study
☐	☒ Antibodies
☐	☒ Eukaryotic cell lines
☒	☐ Palaeontology and archaeology
☐	☒ Animals and other organisms
☒	☐ Clinical data
☒	☐ Dual use research of concern
☒	☐ Plants

Methods

n/a	Involved in the study
☒	☐ ChIP-seq
☐	☒ Flow cytometry
☒	☐ MRI-based neuroimaging

Antibodies

Antibodies used

Brilliant Violet 785TM anti-mouse CD8a [clone 53-6.7 , 100750 BioLegend], Brilliant Violet 650TM anti-mouse/human CD45R/B220 [clone RA3-6B , 103241 biolegend], Brilliant Violet 421TM anti-mouse CD366 (Tim-3) [Clone B8.2C12 , 134019 biolegend], APC/Cyanine7 anti-mouse CD3 [Clone 17A2 , 100221 biolegend], APC/Cyanine7 anti-human CD19 [clone HIB19 , 302218 biolegend], Myc-Tag (9B11) Mouse mAb (Alexa Fluor 647 Conjugate) [NA , 2233 Cell Signaling Technology], Brilliant Violet 605™ anti-mouse CD279 (PD-1) Antibody [clone 29F.1A12 , 135220 biolegend], Brilliant Violet 711™ anti-mouse CD223 (LAG-3) Antibody [clone C9B7W , 125243], Spark Blue™ 550 anti-mouse CD3 Antibody [clone 17A2 , 100259], APC anti-mouse CD105 [Clone MJ7/18 , 120413 biolegend], Pacific Blue™ anti-mouse/rat/human CD27 [Clone LG.3A10 , 124217 biolegend], APC anti-mouse CD134 (OX-40) [clone OX-86 , 119413 biolegend], Brilliant Violet 750™ anti-mouse CD4 [clone GK1.5 , 100467 biolegend], NBP1-69962 RFP Antibody [, NBP1-69962 novus biologicals], alexa Fluor 700 anti-human/mouse/rat CD278 (ICOS) [clone C398.4A , 313528 Biolegend], APC/Fire™ 810 anti-mouse CD45 [clone 30-F11 , 103174 Biolegend], APC/Fire™ 750 anti-mouse CD244.2 (2 B4 B6 Alloantigen) [clone m2 B4 , 133516], PerCP anti-mouse/human CD44 [clone IM7 , 103036 BioLegend], PE/Cyanine7™ anti-mouse TIGIT (Vstm3) [clone 1G9 , 142108 BioLegend], Alexa Fluor® 700 anti-mouse Ly-6G Antibody [clone 1A8 , 127621 BioLegend], PE/Cyanine7 anti-mouse CD2 Antibody [clone RM2-5 , 100113 BioLegend], BD OptiBuild™ BV786 Rat Anti-Mouse Siglec-F [clone E50-2440 , 740956 BD Pharmingen], Alexa Fluor™ 532 MHC Class II (I-A/I-E) Monoclonal [clone M5/114.15.2 , 12-5321-82 eBiosciences], PE-Cyanine7 CD11b Monoclonal [clone M1/70 , 45-0112-82 eBiosciences], CD11c APC-eFluor™ 780 [clone N418 , 47-0114-82 Invitrogen], Alexa Fluor™ 700 CD4 Monoclonal [clone GK1.5 , 17-0041-82 eBiosciences], anti-CSF1R [clone AFS98 , BioXCell], Isotype IgG2 [clone 2A3 , BioXCell], Biotinylated anti-CD117 [clones 2B8 , B376226 BioLegend], streptavidin-SAP conjugate [clone IT-27 , 201-151 Advanced Targeting Systems], Brilliant Violet 510™ anti-mouse/rat XCR1 [clone ZET , BioLegend], Brilliant Violet 421™ anti-mouse MERTK (Mer) [clone 2B10C42 , BioLegend], eVolve™ 605 CD45 Monoclonal [clone 30-F11 , eBiosciences], PerCP/Cyanine5.5 anti-mouse CD64 (FcγRI) [clone X54-5/7.1 , BioLegend]

Validation

All antibodies used were titrated or used as recommended by the manufacturer. Species validation and antibody data are available on the manufacturer website. All antibodies used are commercially available.

Eukaryotic cell lines

Policy information about cell lines and Sex and Gender in Research

Cell line source(s)

293T cells, retroviral producer cell lines, and B16-F10 melanomas were obtained from ATCC. KrasG12D/+ p53−/− (KP) adenocarcinoma cell lines were a kind gift of Dr Miriam Merad. gpg29 fibroblasts were a kind gift from Dr Michel Sadelain.

Authentication

COA were provided with cell lines from ATCC. No other authentication was performed. Morphology and properties pertinent to the experiments, for example, antigen expression, were confirmed by cytometry.

Mycoplasma contamination

KP adenocarcinoma and B16-F10 melanoma cells lines were confirmed negative for Mycoplasma

Commonly misidentified lines
(See ICLAC register)

No commonly mid-identified cell lines were used

Animals and other research organisms

Policy information about studies involving animals; ARRIVE guidelines recommended for reporting animal research, and Sex and Gender in Research

Laboratory animals	All experiments were performed under the approved IACUC protocol # TR202500000026. 5-16 week old C57/Bl6 mus musculus strains were used. WT mice purchased from CRL, Batf3-/- (B6.129S(C)-Batf3tm1Kmm/J), Zbtb46DTR (B6(Cg)-Zbtb46tm1(HBEGF)Mnz/J) mice from Jackson labs. As this study uses an orthotopic lung metastasis model, tumor and size could not be measured by external caliper. Tumor burden was monitored by weekly bioluminescence imaging when possible. All in vivo irradiation experiments were performed in female mice to avoid aggression-related injury in mixed-sex housing of randomized cohorts. Both the KP lung adenocarcinoma and B16-F10 melanoma models were established and characterized in female mice accordingly. Sex was therefore not considered as a variable in the study design.
Wild animals	This study did not involve wild animals
Reporting on sex	Due to risk of fighting between male mice following randomization, experiments were done primarily in female mice.
Field-collected samples	This study did not involve samples collected from the field
Ethics oversight	Icahn School of Medicine at Mount Sinai (ISMMS) Institutional Animal Care and Use Committee

Note that full information on the approval of the study protocol must also be provided in the manuscript.

Plants

Seed stocks	*Report on the source of all seed stocks or other plant material used. If applicable, state the seed stock centre and catalogue number. If plant specimens were collected from the field, describe the collection location, date and sampling procedures.*
Novel plant genotypes	*Describe the methods by which all novel plant genotypes were produced. This includes those generated by transgenic approaches, gene editing, chemical/radiation-based mutagenesis and hybridization. For transgenic lines, describe the transformation method, the number of independent lines analyzed and the generation upon which experiments were performed. For gene-edited lines, describe the editor used, the endogenous sequence targeted for editing, the targeting guide RNA sequence (if applicable) and how the editor was applied.*
Authentication	*Describe any authentication procedures for each seed stock used or novel genotype generated. Describe any experiments used to assess the effect of a mutation and, where applicable, how potential secondary effects (e.g. second site T-DNA insertions, mosiacism, off-target gene editing) were examined.*

Flow Cytometry

Plots

Confirm that:

☒ The axis labels state the marker and fluorochrome used (e.g. CD4-FITC).

☒ The axis scales are clearly visible. Include numbers along axes only for bottom left plot of group (a 'group' is an analysis of identical markers).

☒ All plots are contour plots with outliers or pseudocolor plots.

☒ A numerical value for number of cells or percentage (with statistics) is provided.

Methodology

Sample preparation	Sample preparation is described in detail in Material and Methods. In summary, PBS-perfused Tumor bearing lungs were manually chopped and enzymatically digested with collagenase for 30 minutes at 30C. Cells were then RBC lysed, filtered, and counted prior to staining with antibody cocktails in PBS/EDTA/BSA buffer.
Instrument	Cytek Aurura 5L or BD Fortessa
Software	FlowJo v10.1 (BD), BD FACSDIVA
Cell population abundance	Purity was confirmed at 99% in post-sort samples. Post-sorted cells were contained within the prescribed sorting gates with 95-99%.
Gating strategy	Gating strategies are provided in the figures or figure legends. Isotype stained, or fluorophore-negative samples were used to define positive/negative gating, as indicated in the figure legends.

☒ Tick this box to confirm that a figure exemplifying the gating strategy is provided in the Supplementary Information.
