# Supplementary material for: Tumor irradiation promotes antigen dressing of dendritic cells to enhance CAR T cell persistence and efficacy in lung metastases
Source: Nat Cancer. 2026 May 22;7(7):1104–20. doi: 10.1038/s43018-026-01167-6 (PMC13400301; doi:10.1038/s43018-026-01167-6)
Supplement: Supplementary file 2 — Peer Review File [file 43018_2026_1167_MOESM2_ESM.pdf]

nature portfolio

Peer Review File

Tumor irradiation promotes antigen dressing of dendritic cells to enhance CAR T cell persistence and efficacy in lung metastases

Corresponding Author: Dr Jalal Ahmed

This manuscript has been previously reviewed at another journal. This document only contains information relating to versions considered at Nature Cancer.

This file contains all reviewer reports in order by version, followed by all author rebuttals in order by version.

Version 1:

Reviewer comments:

Reviewer #4

(Remarks to the Author)
General assessment

This manuscript addresses one of the most persistent and clinically relevant limitations of CAR T cell therapy: the failure of CAR T cells to persist and mediate durable tumour control in solid malignancies. Using well-controlled syngeneic models of lung adenocarcinoma and melanoma, the authors demonstrate that local tumour irradiation markedly enhances CAR T cell persistence and therapeutic efficacy through a dendritic cell (DC)–dependent mechanism. The central conceptual advance is the identification of irradiated tumour–conditioned DCs as critical intermediaries that sustain CAR T cells via chimeric receptor engagement, a process the authors term antigen-dressing.

The work is technically strong, logically coherent, and addresses an important unmet need at the interface of radiation oncology and cellular immunotherapy. The data are convincing, and the requirement for DCs is well supported by in vivo depletion, ex vivo functional assays, and transcriptional profiling. Overall, the study represents a meaningful advance for the field and is potentially suitable for publication in Nature Cancer.

That said, several aspects of the manuscript would benefit from deeper mechanistic framing, particularly with respect to radiation biology, immune metabolism, and clinical translation. These issues do not undermine the core findings but should be addressed to maximize impact and generalizability.

Major comments
1. Radiotherapy is positioned as a trigger, but its biological role requires clearer articulation

Radiotherapy is central to the study’s conclusions, yet its biological effects are largely treated as a binary variable (irradiated vs non-irradiated). From a radiation biology perspective, the manuscript would benefit from a more explicit discussion of what irradiation is doing to the tumour–DC axis.

In particular, it remains unclear whether irradiation primarily:

Enhances DC access to intact membrane antigens,

Licenses DCs through DAMP and stress-sensing pathways,

Alters antigen processing or retention at the DC surface,

Or enforces spatial proximity between tumour cells, DCs, and CAR T cells.

While the revised discussion briefly mentions DAMP receptors, this treatment remains superficial given the centrality of RT to the proposed mechanism. The reader is left with a phenomenological association rather than a clearly articulated radiobiological model.

Recommendation:
Reframe radiotherapy not simply as an enabling condition, but as an active immune-conditioning intervention that alters antigen handling, APC function, and spatial immune organization within tumours.

2. DC-dependent CAR T persistence is compelling but mechanistically incomplete

The requirement for dendritic cells is convincingly demonstrated through depletion models and functional assays. However, the manuscript stops short of clarifying what DCs provide beyond antigen display.

Key unresolved questions include:

Whether costimulatory signals (e.g. CD80/CD86) are required for CAR-mediated expansion,

Whether DC-derived cytokines contribute to CAR T survival and persistence,

The relative contribution of DC1 versus DC2 subsets beyond Batf3 dependency.

Given that macrophage depletion fails to phenocopy the effects of irradiation, the importance of DC-specific functions becomes even more pronounced.

Recommendation:
Even if additional experiments are beyond scope, the Discussion should more clearly distinguish what is shown (DC requirement) from what is hypothesized (nature of DC-derived signals), and articulate testable models.

3. Integration with emerging literature on radiotherapy–CAR T synergy is incomplete

The manuscript would be strengthened by more explicitly situating its findings within the rapidly evolving literature on radiotherapy–CAR T interactions. In particular, a recent Nature Cancer study (Ni et al., 2025) demonstrates that FLASH radiotherapy enhances CAR T efficacy through immune–metabolic reprogramming, notably via alterations in myeloid cell lipid metabolism and function.

While the mechanisms described differ—macrophage-centered metabolic rewiring in the FLASH study versus DC-dependent antigen presentation here—the conceptual convergence is important. Together, these studies suggest that radiotherapy enhances CAR T cell therapy through multiple, non-redundant immune mechanisms, including APC licensing and metabolic reprogramming.

At present, the manuscript underutilizes this opportunity to frame radiotherapy as a broader immune-modulating platform rather than a single mechanistic solution.

Recommendation:
Explicitly reference and discuss the FLASH radiotherapy study, positioning the current findings as complementary rather than overlapping, and emphasizing that distinct RT modalities may converge on CAR T support through different immune pathways.

4. Metabolic reprogramming is described but its role remains ambiguous

Transcriptomic analyses show enhanced oxidative phosphorylation and mitochondrial fitness in CAR T cells isolated from irradiated tumours. These data are intriguing, but the causal relationship remains unclear.

The anti-CSF1R experiments convincingly demonstrate that depletion of dominant glucose-consuming macrophages is insufficient to recapitulate irradiation-induced CAR T persistence. This argues against a purely metabolic explanation, yet the manuscript stops short of integrating this result into a coherent model.

Recommendation:
Clarify whether metabolic reprogramming is best interpreted as a downstream consequence of sustained CAR–DC engagement rather than a primary driver, and articulate this distinction more explicitly.

5. Dose, fractionation, and spatial considerations require clearer translational framing

The choice of single-fraction 8 Gy irradiation is reasonable and well justified experimentally. However, the translational implications remain underdeveloped. The data strongly suggest that:

Local, tumour-directed irradiation is required,

Low-dose regimens and total body irradiation are insufficient,

CAR T persistence is spatially restricted to irradiated tumour-bearing tissue.

These observations imply that immune-conditioning by RT may represent a distinct radiobiological endpoint, separable from tumour control.

Recommendation:
Add a focused discussion on dose, fractionation, and irradiated volume, explicitly addressing how these findings might translate to hypofractionated or conformal regimens in patients.

Minor comments

The term “antigen-dressing” is useful but should be more clearly distinguished from classical trogocytosis early in the manuscript.

A schematic summarizing RT-induced tumour stress → DC antigen acquisition → CAR T synapse formation would improve clarity.

Pulmonary toxicity considerations are appropriately acknowledged but could be better integrated into the translational discussion.

Overall recommendation

This is a high-quality, conceptually important manuscript that advances understanding of why CAR T cell therapies fail in solid tumours and how radiotherapy can overcome this limitation. The identification of DC-dependent CAR T persistence in irradiated tumours represents a meaningful contribution to the field.

With revisions that strengthen the radiation biology framework, integrate emerging FLASH radiotherapy literature, and sharpen translational interpretation, the manuscript would meet the standard for publication in Nature Cancer. The recommended changes are primarily conceptual and interpretative rather than experimental, and a revised submission is encouraged.

Reviewer #5

(Remarks to the Author)
This is an elegant study with a strong impact on the field. The authors have addressed the majority of my previous concerns by either providing additional data, editing the manuscript or clarifying certain points. Nevertheless, there remains two points which require attention.

Point 1:
Strong evidence of the dependence on DCs for CART persistence following RT has been provided. There is also robust in vivo data showing that DCs can retain tumour native antigen on their surface, which can directly elicit CART survival/expansion in vitro. As the authors highlighted the number of DCs in these tumours is small, which would significantly reduce the chances of a DC interacting with a CAR T cell in vivo when compared to an in vitro system. An interaction between DCs bearing native antigen and a cognate CART cell could be easily forced in an in vitro setting where only two cell types are present - without the complexity of the TME. Figure 3 shows that DC depletion impacts CART cells only at later timepoints (beyond day 9), which raises the possibility that antigen retained by DCs is crucial when there is less tumour antigen in the environment. Therefore, there is the chance that DCs are only serving as a source of antigen for other cells to present antigen to CAR T cells. The fact that macrophage bearing tumour antigen could not induce CART expansion and that malignant B cells alone can, strengthen the hypothesis of a direct interaction between DCs and CAR T cells in vivo. However, no direct in vivo evidence of this interaction has been provided.
The purpose of using calcium flux (previously suggested) is to confirm synapse formation in vivo. mCherry-labeled DCs would be used alongside fluorescently labelled CART cells. Through intravital imaging, both DCs and CART cells can be tracked, and when they come into close proximity, calcium flux will serve as an indicator that the two cells have interacted and formed a synapse. This approach has been used before to confirm interactions between DCs and B cells, for example (PMID: 16778060). Alternatively, the velocity of cells can be tracked. Immune cells upon interaction present reduced velocity, which can serve as an indicator of interactions. An easier but less conclusive option would be confocal imaging to at least show colocalization of DCs and CART cells in vivo. The confocal image provided by the authors only shows an in vitro interaction. If the authors are unable to provide these experiments due to complexity and extensive time required, I suggest that the title and text should be carefully revised and the conclusion limited to not convey the idea of an in vivo synapse between DCs and tumour cells. In this case, I suggest removing “…via chimeric synapses” from the title and reviewing the text.
Point 2:
In my opinion, the authors have not provided sufficient evidence that DCs are acquiring tumour antigen by trogocytosis. The mechanisms employed by DCs to acquire tumour antigen in this study has not been fully explored and may be a topic of future work.
It is unclear what the receptor/ligands are for tumour cells and DCs to interact in vivo and the purpose of such interactions. Furthermore, the authors cannot exclude the possibility that DCs are retaining native antigen on their surface via receptor-mediated binding. In their rebuttal, the authors argue that such surface display would require antigen internalization followed

by recycling, a process they suggest would take several hours. However, prior studies demonstrate that a certain amount of targeted intact native antigen can be displayed on the surface of cDC1 without requiring trafficking through late endosomal or lysosomal compartments prior to surface presentation (PMID: 32839238). The mechanisms by which DCs retain native antigen over prolonged periods remain incompletely understood, with ongoing debate as to whether this reflects stable surface retention or continuous recycling. As such, the contribution of surface-associated native antigen to the observed effects cannot be formally excluded. Could the authors revise the text to include trogocytosis only as a potential mechanism?

Reviewer #6

(Remarks to the Author)
I co-reviewed this manuscript with one of the reviewers who provided the listed reports. This is part of the Nature Cancer initiative to facilitate training in peer review and to provide appropriate recognition for Early Career Researchers who co-review manuscripts.

Open Access This Peer Review File is licensed under a Creative Commons Attribution 4.0 International License, which permits use, sharing, adaptation, distribution and reproduction in any medium or format, as long as you give appropriate credit to the original author(s) and the source, provide a link to the Creative Commons license, and indicate if changes were made.
In cases where reviewers are anonymous, credit should be given to 'Anonymous Referee' and the source.
The images or other third party material in this Peer Review File are included in the article’s Creative Commons license, unless indicated otherwise in a credit line to the material. If material is not included in the article’s Creative Commons license and your intended use is not permitted by statutory regulation or exceeds the permitted use, you will need to obtain permission directly from the copyright holder.
To view a copy of this license, visit https://creativecommons.org/licenses/by/4.0/

Reviewers' Comments:

Reviewer #4 (Remarks to the Author):

General assessment

This manuscript addresses one of the most persistent and clinically relevant limitations of CAR T cell therapy: the failure of CAR T cells to persist and mediate durable tumour control in solid malignancies. Using well-controlled syngeneic models of lung adenocarcinoma and melanoma, the authors demonstrate that local tumour irradiation markedly enhances CAR T cell persistence and therapeutic efficacy through a dendritic cell (DC)–dependent

mechanism. The central conceptual advance is the identification of irradiated tumour–conditioned DCs as critical intermediaries that sustain CAR T cells via chimeric receptor engagement, a process the authors term antigen-dressing.

The work is technically strong, logically coherent, and addresses an important unmet need at the interface of radiation oncology and cellular immunotherapy. The data are convincing, and the requirement for DCs is well supported by in vivo depletion, ex vivo functional assays, and transcriptional profiling. Overall, the study represents a meaningful advance for the field and is potentially suitable for publication in Nature Cancer.

That said, several aspects of the manuscript would benefit from deeper mechanistic framing, particularly with respect to radiation biology, immune metabolism, and clinical translation. These issues do not undermine the core findings but should be addressed to maximize impact and generalizability.

Major comments
1. Radiotherapy is positioned as a trigger, but its biological role requires clearer articulation

Radiotherapy is central to the study's conclusions, yet its biological effects are largely treated as a binary variable (irradiated vs non-irradiated). From a radiation biology perspective, the manuscript would benefit from a more explicit discussion of what irradiation is doing to the tumour–DC axis.

In particular, it remains unclear whether irradiation primarily:

Enhances DC access to intact membrane antigens,

Licenses DCs through DAMP and stress-sensing pathways,

Alters antigen processing or retention at the DC surface,

Or enforces spatial proximity between tumour cells, DCs, and CAR T cells.

While the revised discussion briefly mentions DAMP receptors, this treatment remains superficial given the centrality of RT to the proposed mechanism. The reader is left with a phenomenological association rather than a clearly articulated radiobiological model.

Recommendation:
Reframe radiotherapy not simply as an enabling condition, but as an active immune-conditioning intervention that alters antigen handling, APC function, and spatial immune organization within tumours.

Thank you for insightful framing. We have redrafted the second paragraph of the discussion as follows:

Here, we resolve how distinct effects of tumor irradiation overcome key barriers to CAR T cell therapy in orthotopic murine models of extensive metastatic lung adenocarcinoma and melanoma. Our data support an updated radiobiological model in which irradiation acts through interconnected mechanisms. First, irradiation conditions tumor cells for antigen transfer to DCs (Fig. 4f, Extended Data Fig. 5e). Second, irradiation licenses productive DC-CAR T cell engagement: DC depletion impacted CAR T cell persistence only in irradiated tumors (Fig. 3a), indicating that this interaction is functionally absent without radiation. Third, CAR T cells expanded selectively within irradiated tumors but not in adjacent normal tissue where the same antigen was widely expressed (Fig. 6f,g,k,l), suggesting that differential effects of irradiation on tumor versus normal tissue drive spatially restricted DC:CAR T cell organization. Critically, sustained CAR T cell persistence required an intact DC compartment. In its absence, tumors relapsed despite strong initial responses (Fig. 3a, Fig. 5e-g), underscoring that durable tumor control depends on DC-mediated immune conditioning.

2. DC-dependent CAR T persistence is compelling but mechanistically incomplete

The requirement for dendritic cells is convincingly demonstrated through depletion models and functional assays. However, the manuscript stops short of clarifying what DCs provide beyond antigen display.

Key unresolved questions include:

Whether costimulatory signals (e.g. CD80/CD86) are required for CAR-mediated expansion,

Whether DC-derived cytokines contribute to CAR T survival and persistence,

The relative contribution of DC1 versus DC2 subsets beyond Batf3 dependency.

Given that macrophage depletion fails to phenocopy the effects of irradiation, the importance of DC-specific functions becomes even more pronounced.

Recommendation:
Even if additional experiments are beyond scope, the Discussion should more clearly distinguish what is shown (DC requirement) from what is hypothesized (nature of DC-derived signals), and articulate testable models.

Thank you again for insightful framing. We have redrafted the penultimate paragraph of the discussion as follows:

While our data establish that DCs are required for sustained CAR T cell persistence in irradiated tumors, the precise signals DCs provide beyond antigen display remain to be defined. Second-generation CARs incorporate costimulatory domains, yet the failure of antigen-dressed macrophages to expand CAR T cells (Extended Data Fig. 5h-i) suggests additional DC-specific signals are required. Several non-mutually exclusive possibilities include DC-specific costimulatory ligands or cytokines that integrate with CAR signaling to sustain proliferation. Moreover, the differential contributions of DC subsets merit consideration: while DC2s drove greater CAR T cell expansion *ex vivo* (Fig. 4c-d), DC1s were essential for durable persistence *in vivo* (Fig. 5f-g), suggesting that these subsets provide complementary signals. Lastly, these data do not exclude the role of critical signals from other sources. Notably, the enrichment of IL-2 signaling pathways observed in CAR T cells from irradiated tumors (Figure 2b, Extended Data Fig. 2c) implicates this essential axis, potentially via autocrine or paracrine signaling. Future studies (CRISPR screening, RNA sequencing) may further elucidate DC-specific signals driving the effects reported here.

3. Integration with emerging literature on radiotherapy–CAR T synergy is incomplete

The manuscript would be strengthened by more explicitly situating its findings within the rapidly evolving literature on radiotherapy–CAR T interactions. In particular, a recent Nature Cancer study (Ni et al., 2025) demonstrates that FLASH radiotherapy enhances CAR T efficacy through immune–metabolic reprogramming, notably via alterations in myeloid cell lipid metabolism and function.

While the mechanisms described differ—macrophage-centered metabolic rewiring in the FLASH study versus DC-dependent antigen presentation here—the conceptual convergence is important. Together, these studies suggest that radiotherapy enhances CAR T cell therapy through multiple, non-redundant immune mechanisms, including APC licensing and metabolic reprogramming.

At present, the manuscript underutilizes this opportunity to frame radiotherapy as a broader immune-modulating platform rather than a single mechanistic solution.

Recommendation:
Explicitly reference and discuss the FLASH radiotherapy study, positioning the current findings as complementary rather than overlapping, and emphasizing that distinct RT modalities may converge on CAR T support through different immune pathways.

We appreciate the opportunity to highlight this manuscript. We have updated the discussion with reference to the study as below:

Other pleiotropic effects of irradiation including altered tumor metabolic activity are not excluded. Doses of radiotherapy >5Gy reduce tumor uptake of tracer-labelled glucose 5-6 days after irradiation67–70. These data suggest that tumor metabolic activity is significantly reduced at the time that we administer CAR T cells. The upregulation of metabolic pathways in CAR T cells in irradiated tumors (Extended Data Fig. 2a,c) may reflect increased availability of glucose, fatty acids71 and other metabolites in irradiated tumors. Further study of the metabolic impact of irradiation on tumor may aid the effective clinical translation of this approach, for example, whether alternative hypofractionated regimens that achieve equivalent metabolic endpoints to a single 8 Gy fraction might yield comparable therapeutic effects. Notably, recent work demonstrates that ultrahigh-dose-rate (>80 Gy s−1) radiotherapy enhances CAR T cell efficacy through macrophage-centered metabolic reprogramming72, though durable CAR T cell persistence was not observed. The sustained CAR T cell numbers seen here with conventional dose-rates (2.1 Gy min-1) may reflect the inclusion of lymphodepleting chemotherapy in the conditioning regimen, as well as the distinct immune microenvironments of brain versus lung tumors. Notwithstanding the effects of irradiation on tumor metabolism, the lack of persistence in Batf3–/– mice despite equivalent irradiation highlights the primacy of DC-mediated immunology to the findings presented.

4. Metabolic reprogramming is described but its role remains ambiguous

Transcriptomic analyses show enhanced oxidative phosphorylation and mitochondrial fitness in CAR T cells isolated from irradiated tumours. These data are intriguing, but the causal relationship remains unclear.

The anti-CSF1R experiments convincingly demonstrate that depletion of dominant glucose-consuming macrophages is insufficient to recapitulate irradiation-induced CAR T persistence. This argues against a purely metabolic explanation, yet the manuscript stops short of integrating this result into a coherent model.

Recommendation:
Clarify whether metabolic reprogramming is best interpreted as a downstream consequence of sustained CAR–DC engagement rather than a primary driver, and articulate this distinction more explicitly.

Thank you for the insightful suggestion. We have added the following paragraph to the discussion:

The lack of persistence in Batf3–/– mice despite equivalent irradiation highlights the primacy of DC-mediated immunology to the findings presented. However, DC-mediated activation of T cells increases their metabolic demands73 , and the metabolic gene signatures observed in CAR T cells from irradiated tumors may therefore reflect the downstream

consequences of sustained DC engagement rather than an independent effect of irradiation on nutrient availability. Consistent with this interpretation, depletion of macrophages, a dominant glucose consumer in the TME, was insufficient to enhance CAR T cell persistence (Extended Data Fig. 3). Rather, irradiation may create a metabolically permissive microenvironment that accommodates the heightened demands of DC-activated CAR T cells.

5. Dose, fractionation, and spatial considerations require clearer translational framing

The choice of single-fraction 8 Gy irradiation is reasonable and well justified experimentally. However, the translational implications remain underdeveloped. The data strongly suggest that:

Local, tumour-directed irradiation is required,

Low-dose regimens and total body irradiation are insufficient,

CAR T persistence is spatially restricted to irradiated tumour-bearing tissue.

These observations imply that immune-conditioning by RT may represent a distinct radiobiological endpoint, separable from tumour control.

Recommendation:
Add a focused discussion on dose, fractionation, and irradiated volume, explicitly addressing how these findings might translate to hypofractionated or conformal regimens in patients.

Thank you for the thoughtful point. We have integrated these together with the Minor Comment on “Pulmonary toxicity considerations” into a compiled translational paragraph in the discussion, as below:

While further studies are needed to optimize this strategy for the clinic, our data provide important guideposts. Low doses of focal or total body irradiation did not enhance CAR T cell persistence in our models of extensive lung metastases. Although prior work has shown that low-dose local radiotherapy (RT)21 or low-dose TBI22 can enhance CAR T cell efficacy against hematologic cancers in murine models, and there is an active clinical trial investigating low-dose RT to bridge patients to CAR T cells for patients with large B-cell lymphoma55, our data and prior work56 suggest that low-dose RT may not work in the context of histologies such as NSCLC or melanoma, which are amongst the most radioresistant24, in contrast to lymphoid lineages which are radiosensitive23 (Extended Data Fig. 1j). These findings suggest that the capacity of radiation to facilitate DC:CAR T cell interactions is a distinct radiobiological endpoint. Hypofractionated conformal regimens may be ideally suited to achieve this in patients. Whether RT parameters should be

optimized for immune-conditioning warrants prospective investigation. To irradiate the widely disseminated advanced lesions in our lung metastasis model, we employed Anterior-Posterior fields to treat the bilateral lungs with doses not associated with radiation-induced pulmonary toxicity in mice or non-human primates18. However, recent data suggest that tumor-directed hypofractionated radiotherapy is sufficient to enhance immunotherapy for NSCLC65,66. Thus, while not specifically tested in our study, we speculate that conformal tumor-directed radiotherapy that spares uninvolved tissue can achieve the same effects reported here, and mitigate risks of pulmonary toxicity.

Minor comments

The term “antigen-dressing” is useful but should be more clearly distinguished from classical trogocytosis early in the manuscript.

We refer to “Trogocytic antigen-dressing” to make the argument that cell-surface antigens were transferred via a trogocytic process, since trypsinization abrogated this effect.

A schematic summarizing RT-induced tumour stress → DC antigen acquisition → CAR T synapse formation would improve clarity.
Please see the example schema below:

Figure Redacted

Pulmonary toxicity considerations are appropriately acknowledged but could be better integrated into the translational discussion.
See response to Major Comment 5.

Overall recommendation

This is a high-quality, conceptually important manuscript that advances understanding of why CAR T cell therapies fail in solid tumours and how radiotherapy can overcome this limitation. The identification of DC-dependent CAR T persistence in irradiated tumours represents a meaningful contribution to the field.

With revisions that strengthen the radiation biology framework, integrate emerging FLASH radiotherapy literature, and sharpen translational interpretation, the manuscript would meet the standard for publication in Nature Cancer. The recommended changes are primarily conceptual and interpretative rather than experimental, and a revised submission is encouraged.

Thank you very much.

Reviewer #5 (Remarks to the Author):

This is an elegant study with a strong impact on the field. The authors have addressed the majority of my previous concerns by either providing additional data, editing the manuscript or clarifying certain points. Nevertheless, there remains two points which require attention.

Point 1:
Strong evidence of the dependence on DCs for CART persistence following RT has been provided. There is also robust in vivo data showing that DCs can retain tumour native antigen on their surface, which can directly elicit CART survival/expansion in vitro. As the authors highlighted the number of DCs in these tumours is small, which would significantly reduce the chances of a DC interacting with a CAR T cell in vivo when compared to an in vitro system. An interaction between DCs bearing native antigen and a cognate CART cell could be easily forced in an in vitro setting where only two cell types are present - without the complexity of the TME. Figure 3 shows that DC depletion impacts CART cells only at later timepoints (beyond day 9), which raises the possibility that antigen retained by DCs is crucial when there is less tumour antigen in the environment. Therefore, there is the chance that DCs are only serving as a source of antigen for other cells to present antigen to CAR T cells. The fact that macrophage bearing tumour antigen could not induce CART expansion and that malignant B cells alone can, strengthen the hypothesis of a direct interaction between DCs and CAR T cells in vivo. However, no direct in vivo evidence of this interaction has been provided.
The purpose of using calcium flux (previously suggested) is to confirm synapse formation in vivo. mCherry-labeled DCs would be used alongside fluorescently labelled CART cells. Through intravital imaging, both DCs and CART cells can be tracked, and when they come

into close proximity, calcium flux will serve as an indicator that the two cells have interacted and formed a synapse. This approach has been used before to confirm interactions between DCs and B cells, for example (PMID: 16778060). Alternatively, the velocity of cells can be tracked. Immune cells upon interaction present reduced velocity, which can serve as an indicator of interactions. An easier but less conclusive option would be confocal imaging to at least show colocalization of DCs and CART cells in vivo. The confocal image provided by the authors only shows an in vitro interaction. If the authors are unable to provide these experiments due to complexity and extensive time required, I suggest that the title and text should be carefully revised and the conclusion limited to not convey the idea of an in vivo synapse between DCs and tumour cells. In this case, I suggest removing "…via chimeric synapses" from the title and reviewing the text.

We appreciate the reviewers point that "chimeric synapse" implies direct imaging evidence of synapse formation that wasn't obtained. We have removed "chimeric synapses" from the title and text. We have also reread the text and could not find an instance where we suggest that there is an "in vivo synapse between DCs and tumour cells", though this was likely a typo and means to say "synapse between DCs and CAR T cells". If this is not the case, we can address where there might be ambiguity in the text if provided with details where this was noted.

Point 2:
In my opinion, the authors have not provided sufficient evidence that DCs are acquiring tumour antigen by trogocytosis. The mechanisms employed by DCs to acquire tumour antigen in this study has not been fully explored and may be a topic of future work.
It is unclear what the receptor/ligands are for tumour cells and DCs to interact in vivo and the purpose of such interactions. Furthermore, the authors cannot exclude the possibility that DCs are retaining native antigen on their surface via receptor-mediated binding. In their rebuttal, the authors argue that such surface display would require antigen internalization followed by recycling, a process they suggest would take several hours. However, prior studies demonstrate that a certain amount of targeted intact native antigen can be displayed on the surface of cDC1 without requiring trafficking through late endosomal or lysosomal compartments prior to surface presentation (PMID: 32839238). The mechanisms by which DCs retain native antigen over prolonged periods remain incompletely understood, with ongoing debate as to whether this reflects stable surface retention or continuous recycling. As such, the contribution of surface-associated native antigen to the observed effects cannot be formally excluded. Could the authors revise the text to include trogocytosis only as a potential mechanism?

Thank you for the thoughtful discussion.
The referee provided the citation by Kato et al. (PMID: 32839238), which showed that DC1s present **Clec9A-targeted native antigen** for B cell activation. As noted by the reviewer,

Kato et al. targeted antigens to DCs by coupling antigens to anti-Clec9 antibody, which were then delivered to DCs. The Clec9A receptor did not bind native antigen, rather, it was labelled by Ag-antibody (akin to a fluorophore-conjugated antibody staining a cell). Thus, the Ag-antibody “decorates” the Clec9A epitope on DCs, and no action to display antigen is taken by the DCs. Therefore, we argue that this study does not show that native antigen obtained via receptor-mediated binding is subsequently displayed by DCs (with or without trafficking through late endosomal or lysosomal compartments).

In contrast, we found that DCs took up antigen directly from tumor cells, and did so within a time frame that is consistent with a trogocytic process. Critically, we found that trypsinization of tumor cells prior to coculture with BMDCs abrogated antigen-dressing. This demonstrates that trypsin sensitive moieties on the tumor cell surface**, and not hCD19 antigen,** provide a substrate for DC antigen-dressing (hCD19 is not trypsin-sensitive - Extended data Fig1). If DCs are retaining native hCD19 antigen on their surface with receptor-mediated binding, then trypsinization would not be expected to impact the amount of receptor-bound antigen on DCs, since hCD19 molecules on tumor are not impacted by trypsinization (Extended data Fig1).

We recognize that the mechanisms employed by DCs to obtain tumor-derived target antigens have not been fully explored, though these data together are more in line with a trogocytic process. We recognize that other mechanisms may also contribute, and have updated the discussion to note the following:

“As professional phagocytes, DCs express diverse families of receptors that recognize damaged and dead cells (adhesion receptors, TAM and scavenger receptors, C-type lectin receptors) that may contribute to antigen-dressing, and we do not exclude processes other than trogocytosis that may contribute to the display of native tumor antigen on DCs”

Reviewer #6 (Remarks to the Author):

I co-reviewed this manuscript with one of the reviewers who provided the listed reports. This is part of the Nature Cancer initiative to facilitate training in peer review and to provide appropriate recognition for Early Career Researchers who co-review manuscripts.

This email has been sent through the Springer Nature Tracking System NY-610A-NPG&MTS

Confidentiality Statement:

This e-mail is confidential and subject to copyright. Any unauthorised use or disclosure of its contents is prohibited. If you have received this email in error please notify our Springer Nature Tracking System Helpdesk team at http://platformsupport.nature.com .

Details of the confidentiality and pre-publicity policy may be found here http://www.nature.com/authors/policies/confidentiality.html

Privacy Policy | Update Profile
